# Efficacy of corticosteroids for hand osteoarthritis - a systematic review and meta-analysis of randomized controlled trials

**DOI:** 10.1186/s12891-022-05619-9

**Published:** 2022-07-13

**Authors:** Mahnuma Mahfuz Estee, Flavia M. Cicuttini, Matthew J. Page, Anant D. Butala, Anita E. Wluka, Sultana Monira Hussain, Yuanyuan Wang

**Affiliations:** School of Public Health and Preventive Medicine, Monash University, Melbourne, 553 St Kilda Road, Melbourne, Victoria 3004 Australia

**Keywords:** Hand osteoarthritis, Corticosteroids, Randomized controlled trials, Pain, Function, Oral, Intra-articular injection

## Abstract

**Background:**

There is some evidence that corticosteroids may have a beneficial effect in hand osteoarthritis. We examined the efficacy of corticosteroids on symptoms and structural outcomes in hand osteoarthritis.

**Methods:**

Ovid MEDLINE, Embase and Cochrane Central Register of Controlled Trials were searched from inception to October 2021 for randomized controlled trials investigating the efficacy of corticosteroids in hand osteoarthritis. Two authors independently screened records, extracted data, and assessed risk of bias using the RoB 2 tool. Standardized mean difference (SMD) or mean difference (MD) was calculated, and random-effects meta-analyses were performed.

**Results:**

Of 13 included trials, 3 examined oral corticosteroids and clinical outcomes in any hand joints, 9 examined intra-articular injection of corticosteroids and clinical outcomes at the first carpometacarpal joint and one in the interphalangeal joints. In meta-analysis, oral corticosteroids reduced pain (SMD -0.53, 95% CI -0.79 to -0.28) and improved stiffness (MD -5.03, 95% CI -9.91 to -0.15; Australian Canadian Osteoarthritis Hand Index stiffness subscale) and function (SMD -0.37, 95% CI -0.63 to -0.12) at 4-6 weeks. However, there was no significant persistent effect on pain and function at 3 months which was 6-8 weeks after study medication was stopped. There was no significant effect of intra-articular corticosteroids on pain or function at 4-6 weeks or over 3-12 months in first carpometacarpal osteoarthritis. Two trials evaluated joint structure at 4-6 weeks: one study showed oral corticosteroids reduced synovial thickening, neither showed an effect on synovitis.

**Conclusions:**

There was low-certainty evidence for a medium effect of oral corticosteroids on pain relief and stiffness improvement and small-to-medium effect on functional improvement at 4-6 weeks, with no significant effect for intra-articular corticosteroids. Corticosteroids had no significant effect on any outcomes over longer term (3-12 months) off treatment. No trials examined the effect of corticosteroids on disease progression. The role of corticosteroids in hand osteoarthritis is limited.

**Supplementary Information:**

The online version contains supplementary material available at 10.1186/s12891-022-05619-9.

## Background

Hand osteoarthritis (OA) is a prevalent joint condition, causing disabling pain, reduced mobility, impaired daily functioning and quality of life [1–4]. Hand OA affects at least one hand joint in most people aged over 55 years [5]. Current clinical guidelines recommend topical non-steroidal anti-inflammatory drugs (NSAIDs), oral analgesics, intra-articular corticosteroid injection, and non-pharmacological treatment to manage hand OA [6, 7].

Corticosteroids are anti-inflammatory medications frequently used in musculoskeletal diseases. Oral corticosteroids are effective in treating pain in hand OA with inflammatory features, but their use is restricted due to the systemic side effects [8]. A previous meta-analysis showed that intra-articular corticosteroid injections were no more effective than placebo in improving pain in carpometacarpal OA, with a lack of data on interphalangeal OA, thus precluding conclusions regarding efficacy [9]. No systematic review has evaluated the effect of corticosteroids on structural outcomes in hand OA. Given the uncertainty about their benefit, and to extend previous studies by including more clinical trials assessing the efficacy of corticosteroids in hand OA published after the previous systematic reviews and/or meta-analyses [9–12], we conducted a systematic review and meta-analysis of randomized controlled trials to investigate the efficacy of corticosteroids by any route, on symptoms and structural outcomes in hand OA.

## Methods

The systematic review was conducted in accordance with the Preferred Reporting Items for Systematic Review and Meta-Analysis (PRISMA) guidelines [13]. It was registered on the PROSPERO (CRD42021225694).

### Search strategies

A systematic literature search was performed from inception to October 2021 using Ovid MEDLINE(R) and Epub Ahead of Print, In-Process, In-Data-Review & Other Non-Indexed Citations, Daily and Versions(R), Ovid Embase Classic+Embase, Ovid EBM Reviews - Cochrane Central Register of Controlled Trials. Search terms relating to corticosteroids, hand OA and randomized controlled trials were used (Supplementary Table 1). The references list of included articles and published reviews were searched.

### Trial registry search

US National Institutes of Health Trial Register (http://www.clinicaltrials.gov), European Clinical Trial Register (http://www.clinicaltrialsregister.eu), Australian New Zealand Clinical Trials Registry (http://www.anzctr.org.au), and International Standard Randomised Controlled Trial Number registry (http://www.isrctn.com) were searched for unpublished trials with “Completed” or “Unknown” status that met the eligibility criteria of our systematic review.

### Eligibility criteria

#### Population

Studies of participants diagnosed with OA of the interphalangeal joint, carpometacarpal joint, thumb, and overall hand involvement, based on the American College of Rheumatology (ACR) criteria or other valid criteria (clinical or radiological) were included [14]. Studies including other types of arthritis were excluded.

#### Intervention

Studies with one treatment arm receiving corticosteroid of any generic or tradename, route, dose, duration, frequency and combination form were eligible.

#### Comparator

The comparator was placebo or any other pharmacological or non-pharmacological intervention including combined treatments for hand OA, or with corticosteroids at different doses, durations, and frequencies.

#### Outcome measures

Studies with at least one outcome related to hand OA were eligible. Studies with pain, function and grip strength measured using any instrument as main outcomes were included. We also included studies with other outcomes, e.g. morning stiffness, lateral pinch, tip pinch, pinch strength, chunk pinch, pain intensity on pressure, pain threshold, tenderness, swollen joint count, structural changes/damage, mobility, fulfillment of Osteoarthritis Research Society International (OARSI)/Outcome Measures in Rheumatology (OMERACT) respondent criteria, palpation for joint tenderness, provocative tests (Grind test and Lever test), and patient satisfaction.

Randomized controlled trials, written in English and available in full-text were eligible. We excluded conference abstracts, review articles, protocol papers, animal studies, editorials, observational studies, non-randomized trials, and studies without a comparison group.

### Screening and data extraction

Identified citations were exported to Covidence software. MME and ADB independently screened the title and abstract, and conducted full-text screening to identify eligible studies, with disagreements resolved by YW. MME and ADB independently extracted the data, with disagreements resolved by YW or FMC. Data on demographics (age, sex) and number of participants, definition/description of hand OA, intervention and comparator characteristics (dose, frequency, route of administration, duration of intervention), outcome measures and time points, and results were extracted. When preferred forms of data were unavailable, the study corresponding author was contacted.

### Risk of bias assessment

MME and YW independently assessed the risk of bias using the Cochrane Risk of Bias (RoB) 2 tool [15] with disagreements resolved by MPJ. The results were visualized using Robvis tool [16].

### Data synthesis and reporting

We presented the summary statistics (e.g. means and standard deviations per group) and effect estimates [e.g. mean differences (MD) with 95% confidence intervals (CI)] of each study according to route of administration (oral or intra-articular) for all outcomes (Supplementary Tables 2 and 3). When study characteristics were sufficiently similar (i.e. same route of administration and outcome domain evaluated at a similar time point regardless of dose) and necessary statistics were available, those studies were combined in meta-analysis. We synthesized MDs for studies using the same scale to measure the outcome domain. If different scales were used across studies to measure the same outcome domain, we calculated standardized mean differences (SMD). Where necessary, standard errors of the mean or interquartile ranges were converted to standard deviations using the Cochrane Handbook formulae [17].

For meta-analyses, the effect estimates were synthesized using a random-effects model, assuming that clinical and methodological heterogeneity are likely to exist and have an effect on the results. All meta-analyses were conducted using the inverse-variance method, the DerSimonian and Laird method of moments estimator was used to estimate the between-study variance, with 95% CIs calculated using the Wald type method [18]. Heterogeneity was assessed visually by inspecting the forest plots and by calculating the I^2^ statistic [19]. We did not conduct subgroup or sensitivity analysis. All statistical analyses were performed using the metan package in Stata 16 (College Station, Texas USA).

### Assessment of risk of bias due to missing evidence and certainty in the body of evidence

We assessed risk of bias due to missing evidence (arising from publication bias and selective reporting bias) in the meta-analyses of pain and function at 4-6 weeks, following the framework outlined in the Cochrane Handbook [17]. We assessed certainty in the body of evidence for main comparisons (i.e. oral corticosteroid vs placebo, and intra-articular corticosteroid vs placebo) in pain and function using the GRADE approach [20]. We considered the five standard domains for downgrading evidence in GRADE to inform an overall assessment of certainty for each outcome, which was judged to be high, moderate, low and very low. All assessments were performed by MME and verified by MJP.

## Results

### Study selection

**Fig. 1:**
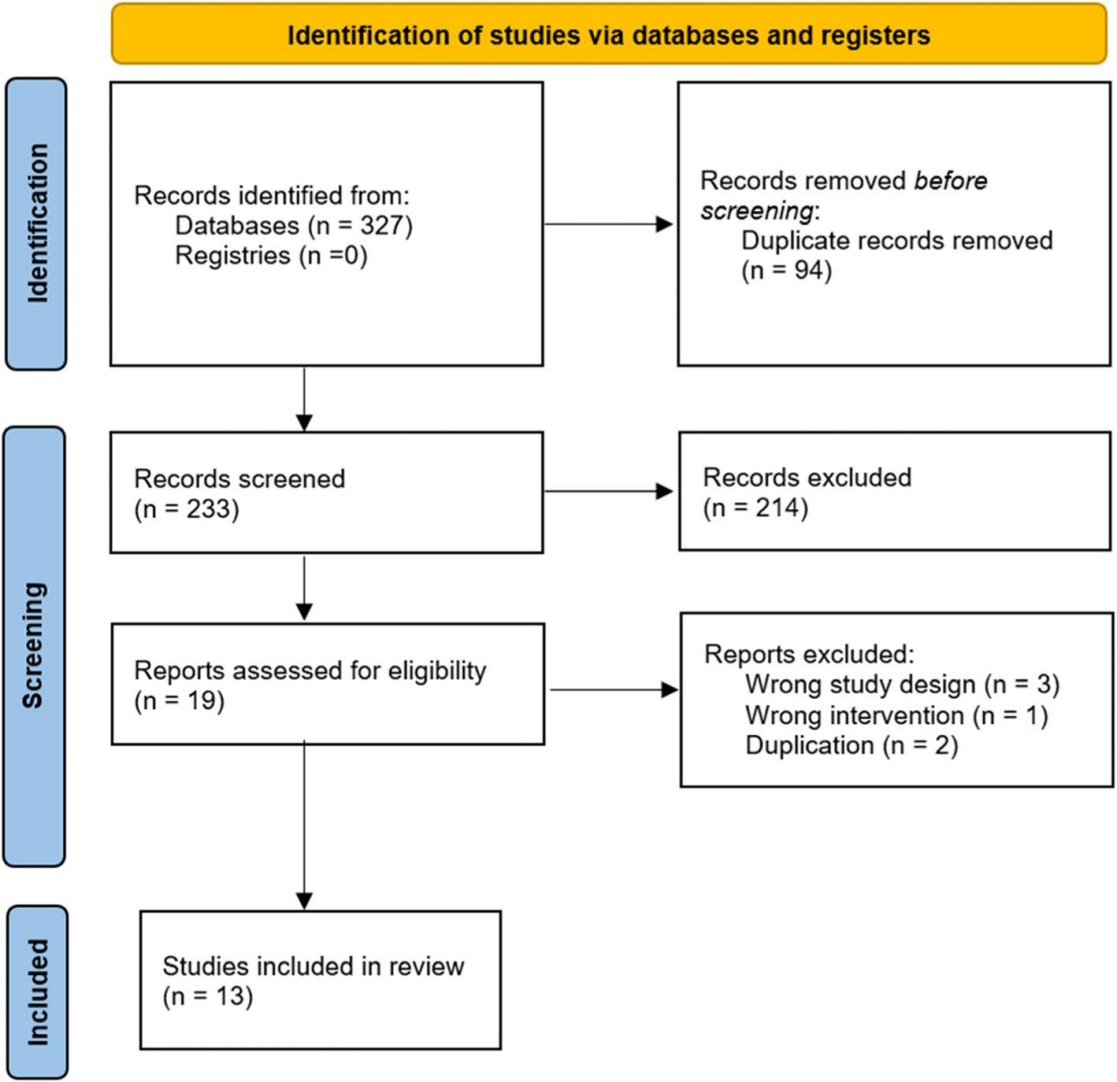
PRISMA flow diagram for efficacy of corticosteroids on hand osteoarthritis

The systematic search retrieved 327 citations. After removing duplicates, 233 articles remained for title and abstract screening, and 19 studies underwent full-text screening. Six studies were excluded, leaving 13 studies eligible for data extraction (Fig. 1). No additional articles were found by searching the references of published research or review articles.

### Trial registry search

Three unpublished trials had an actual or estimated completion date prior to 2021 that were potentially eligible for our systematic review (Supplementary Table 4).

### Overall description of included studies

Table 1 provides an overview of the 13 studies published between 2004 and 2021; all of parallel group design, evaluating a total of 780 participants. Seven studies recruited from outpatient clinic or hospital referred patients [8, 22, 23, 26, 28–30], and six studies did not report the source [21, 24, 25, 27, 31, 32]. The mean age ranged 52.5-63.9 years and proportion of females ranged 73%-100%.

**Table 1:** Characteristics of included studies

	Study Year Country	Study setting	Mean age (years); Number; Female (%)	OA definition and joint location	Intervention and control	Frequency Dose Duration of treatment	Primary outcome	Follow-up
Oral	Kvien, 2008, Norway [21]	Not defined	60.4; 83; 77 (93%)	-ACR; Clinical (>1 swollen joint, >1 tender joint) and radiological (KL score >2) diagnosis -Hand	Prednisolone and dipyridamole vs placebo	1/day; 3mg prednisolone + 200mg dipyridamole -> 1-7 days 1/week; 3mg prednisolone + 400mg dipyridamole ->8-42 days; 6 weeks	Reduction in pain from baseline to day 42 (AUSCAN scale)	7,14,28, 42 days
	Wenham, 2012, UK [22]	Outpatient clinics	61.5; 70; 57 (81.5%)	-ACR; clinical and radiological (KL score ≥1) diagnosis -Hand	Prednisolone vs placebo	1/day; 5mg; 4 weeks	Change in pain at 4 weeks (VAS)	4,12 weeks
	Kroon, 2019, The Netherlands [8]	Outpatient clinics	63.9; 92; 73 (79.5%)	-ACR; clinical diagnosis (signs of inflammation in DIP and PIP joints) -Hand	Prednisolone vs placebo	1/day; 10mg->6 weeks, then 5mg->1 week, then 2.5mg->1week 6 weeks	Change in pain at 6 weeks (VAS)	6,8,14 weeks
Intra-articular	Meenagh, 2004, UK [23]	Hospital referred patients	60; 40; 36 (90%)	-ACR -Thumb CMC	Triamcinolone hexacetonide vs saline	Once; Triamcinolone: 5mg/0.25mL Sterile 0.9% saline: 0.25mL	20% Pain improvement of at 24 weeks (VAS)	4,12,24 weeks
	Stahl, 2005, Israel [24]	Not defined	62; 52; 46 (88.5%)	-Clinical and radiological (EL stage II) diagnosis -Thumb CMC	Methylprednisolone acetate vs hyaluronic acid	1/week; Methylprednisolone: 40mg HA: 15mg; 1 week	Not reported	1,3,6 months
	Fuchs, 2006, Germany [25]	Not defined	Median 61; 56; 45 (80%)	-Clinical and radiological (KL score >0) diagnosis -Thumb CMC	Triamcinolone vs HA	1/week; Triamcinolone: 10mg/1mL HA: 10mg/1mL; 3 weeks	Pain (VAS)	1,2,3,4,5,14,26 weeks
	Heyworth, 2008, USA [26]	Enrolled from the practices of the 2 senior authors	63; 60; 52 (86.7%)	-Standard clinical and radiological diagnosis -Thumb CMC	Betamethasone vs HA vs placebo	1/week; Betamethasone: 1^st^ week-> placebo and 2^nd^ week-> (no dose mentioned)/1mL active component HA: 1mL/week Placebo: 1mL normal saline/week; 2 weeks	Not reported	2,4,12,24 weeks
	Bahadir, 2009, Turkey [27]	Not defined	62; 40; 40 (100%)	-Clinical and radiological (EL stage II-III) diagnosis -TMJ	Triamcinolone vs HA	1/week; Triamcinolone: 20mg/0.5 mL HA: 5mg/0.5mL; 3 weeks	Not reported	1,3,6,12 months
	Jahangiri, 2014, Iran [28]	Clinic, or referred by primary care physicians	63.6; 60; 44 (73%)	-Clinical and radiological (EL stage II-IV) diagnosis -Thumb CMC	Methylprednisolone and lidocaine vs dextrose and lidocaine	1/month; Methylprednisolone: 1^st^ and 2^nd^ month-> 1 mL 0.9% saline, 3^rd^ month-> 40mg methylprednisolone/0.5 ml and 0.5mL 2% lidocaine Dextrose: 1^st^, 2^nd^ and 3^rd^ month-> 20% dextrose /0.5mL and 0.5 mL 2% lidocaine; 3 months	Pain intensity (VAS)	1,2,6 months
	Monfort, 2014, Spain [29]	Outpatient clinics	62.8; 88; 77 (88%)	-ACR, clinical and radiological (KL score 1-3) diagnosis -Thumb CMC	Betamethasone vs HA	1/week; Betamethasone: 3mg/0.5mL HA: 5mg/0.5mL; 3 weeks	Function (FIHOA Score)	7,14,30,90,180 days
	Spolidoro, 2015, Brazil [30]	Outpatient clinic	60.7%; 60; 58 (96.7%)	-ACR, clinical and radiological (osteophytes) diagnosis -IP	Triamcinolone and lidocaine vs lidocaine	1/week; Triamcinolone: 4mg/0.2mL (DIP) or 6mg/0.3mL (PIP) and 2% lidocaine Lidocaine: 0.1mL 2% lidocaine; 1 week	Pain at rest (VAS)	1,4,8,12 weeks
	Sabaah 2020, Egypt [31]	Not defined	52.5; 38 (86.7%)	-Clinical and radiological (EL stage IV) diagnosis -Thumb CMC	Betamethasone and lidocaine vs HA vs PRP	Once; Betamethasone: (dose not mentioned)/1mL betamethasone and 0.25mL lidocaine HA: 1mL PRP: 1mL	Not reported	4,12 weeks
	Malahias, 2021, Greece [32]	Not defined	62.9; 33; 26 (81%)	-Clinical and radiological (EL stage I-III) diagnosis -Thumb CMC	Methylprednisolone and lidocaine vs PRP	1/15days; Methylprednisolone: 125mg/2mL and 2% lidocaine PRP: 2.5mL; 30 days	Not reported	3,12 months

*ACR* American College of Rheumatology, *AUSCAN* Australian Canadian Osteoarthritis Hand Index, *CMC* Carpometacarpal joint, *DIP* Distal interphalangeal joint, *EL* Eaton and Litter classification, *FIHOA* Functional Index for Hand Osteoarthritis, *HA* Hyaluronic acid, *IP* Interphalangeal joint, *KL* Kellgren-Lawrence, *PIP* Proximal interphalangeal joint, *PRP* Platelet-rich plasma, *TMJ* Trapeziometacarpal joint, *VAS* Visual analogue scale

Hand OA was defined by combination of ACR criteria and/or clinical diagnosis along with radiological evidence. Six studies defined OA based on the ACR criteria [8, 21–23, 29, 30] with five of them also including clinical diagnosis [8, 21, 22, 29, 30]. The other seven studies defined OA clinically [24–28, 31, 32], based on the duration and level of pain [25, 28], clinical features [26, 31], or not specified [24, 27, 32]. Two studies evaluated special phenotypes of OA: presence of inflammation [8] and swollen and tender joint [21]. Eleven studies included radiological evidence to define hand OA, using Kellgren-Lawrence and Eaton-Lister classification [21, 22, 24–32]. All three studies of oral corticosteroids examined patients with hand OA in general [8, 21, 22], with two studies excluding patients with predominant or isolated pain at the first carpometacarpal joint [8, 22]. Among the 10 studies of intra-articular corticosteroids, 9 studies examined patients with carpometacarpal OA of the thumb [23–29, 31, 32] and one study examined patients with interphalangeal OA [30].

The duration of follow-up varied from 1 to 12 months; 12 studies had 4-6 weeks [8, 21–31], 12 studies had 3 months [8, 21–27, 29–32], and nine studies had 6-12 months follow-up [23–30, 32].

### Intervention

#### Oral corticosteroids

Three studies evaluated prednisolone versus placebo [8, 21, 22], with doses ranged 3-10 mg and 4-6 weeks treatment duration. Kvien used a combination of prednisolone and dipyridamole [21].

#### Intra-articular corticosteroid injection

Ten studies evaluated intra-articular injection of triamcinolone [23, 25, 27, 30], methylprednisolone [24, 28, 32] and betamethasone [26, 29, 31]. The control arm received platelet-rich plasma [31, 32], hyaluronic acid [24–27, 29, 31], dextrose [28], saline [23], lidocaine [30] or placebo not defined [26]. Three studies used a combination of corticosteroid and lidocaine [30–32]. Six studies used a single injection [23, 24, 26, 28, 30, 31]. Heyworth administered placebo in the 1^st^ week and 1 mL betamethasone in the 2^nd^ week [26]. Jahangiri administered saline in the 1^st^ and 2^nd^ month before administrating methylprednisolone 40mg/0.5mL in the 3^rd^ month [28]. One study performed two 125mg/2mL methylprednisolone injections with a 15 days interval [32], and three studies performed weekly injection of triamcinolone (10mg/1mL and 20mg/0.5mL) [25, 27] and betamethasone (3mg/0.5mL) [29] for 3 weeks. Spolidoro administered triamcinolone 4mg/0.2mL for distal interphalangeal and 6mg/0.3mL for proximal interphalangeal joints [30]. Three out of the 10 studies used imaging guidance (radiography or ultrasound) for intra-articular injections [23, 29, 32].

### Outcome measures

Clinical outcomes included pain [8, 21–32], function [8, 21, 22, 26–32], stiffness [21–23, 30], grip strength [8, 24, 26, 27, 30, 31], lateral pinch [24, 26, 27], tip pinch [26, 27], pinch strength [28, 30, 31], chunk pinch [27], pain intensity on pressure [28], pain threshold [28], tenderness [23], swollen joint count [30], OARSI/OMERACT responder criteria [8], palpation for joint tenderness [31], provocative tests (Grind test and Lever test) [31], and patient satisfaction [32]. Two studies evaluated structural outcomes: synovitis and bone marrow lesions from magnetic resonance imaging (MRI), and synovial thickening and power Doppler signal from ultrasound [8, 22].

### Risk of bias assessment

Considering the risk of bias for pain and function, four studies had low risk of bias [8, 22, 28, 30], six had some concerns [23, 25, 26, 29, 31, 32], and three had high risk of bias [21, 24, 27] (Supplementary Figure 1). Three studies [21, 24, 27] were rated high risk of bias because of deviation from intended intervention, missing outcome data, or bias in outcome measurement. Studies were rated as having some concerns on bias in selection of reported result [21, 23–27, 29, 31, 32] and randomization process [21, 24, 25, 27, 29, 31].

### Short-term (4-6 weeks) effect of corticosteroids on pain

**Fig. 2:**
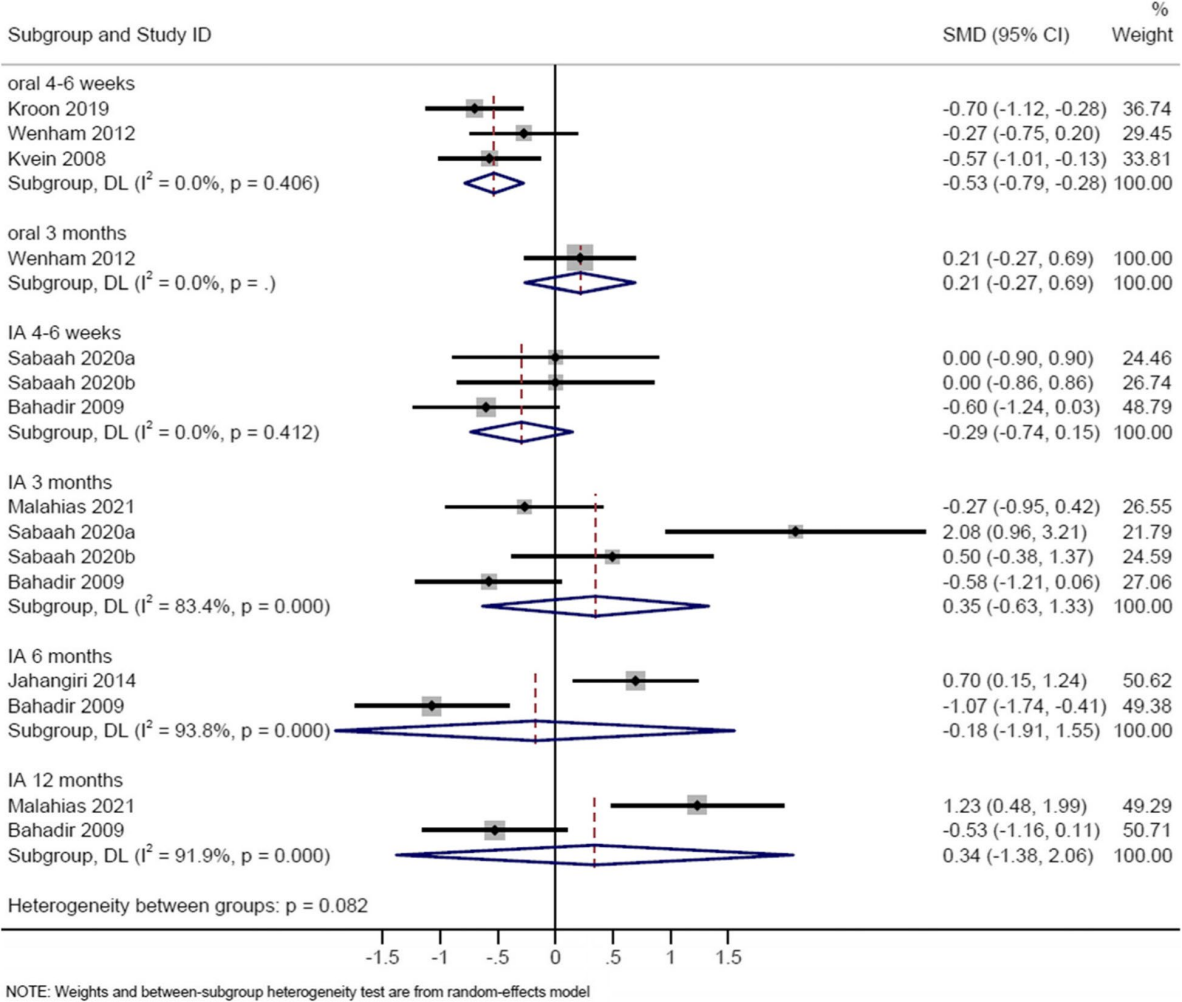
Random-effects meta-analysis of the standard mean difference in pain, corticosteroid vs control on treatment (4-6 weeks) and off treatment follow-up (3-12 months)

Three studies evaluated oral corticosteroids compared to placebo [8, 21, 22] (Table 2; Supplementary Table 2), with meta-analysis demonstrating favourable effect of corticosteroids on reducing pain (SMD -0.53, 95% CI -0.79 to -0.28) (Fig. 2). The result was similar when Kvien’s study [21] was excluded (SMD -0.50, 95% CI -0.92 to -0.08).

**Table 2:** Effect of corticosteroids versus control on pain and function

	Study	Scale	Range	Mean (SD), calculated Corticosteroid	Mean (SD), calculated Control	Number Corticosteroid	Number Control	Mean difference (95% CI)	Meta-analysis (SMD 95% CI)
Short term effect (4-6 weeks)/pain									
Oral	Kroon 2019 [8]*	VAS	0-100	-21.5 (21.7)	-5.2 (24.3)	46	46	-16.5 (-26.1 to -6.9)	-0.53 (-0.79, -0.28)
	Wenham 2012 [22]*	VAS	0-100	-20 (10.8)	-17 (10.8)	35	35	NR	
	Kvien 2008 [21]*	VAS	0-100	-18.6 (21.4)	-6.3 (21.1)	42	41	-12.3 (-21.5 to -3)	
Intra-articular	Sabaah 2020(a) [31]	VAS	0-10	4 (1.6)	4 (1.6)	15	15	NR	MD 0.41 (-1.51, 2.33)
	Sabaah 2020(b) [31]	VAS	0-10	4 (1.6)	4 (1.6)	15	15	NR	
	Jahangiri 2014 [28]	VAS	0-100	NR	NR	29	30	-0.7 (-1.8 to 0.2)	
	Bahadir 2009 [27]	VAS	0-10	3.1 (2.6)	4.4 (2.6)	20	20	NR	
Short term effect (4-6 weeks)/function									
Oral	Kroon 2019 [8]*	AUSCAN	0-100	-6.5 (7.4)	-2.7 (4.7)	46	46	-3.7 (-6.2 to -1.1)	-0.37 (-0.63, -0.12)
	Wenham 2012 [22]*	AUSCAN	0-100	-10 (9.3)	-8 (9.3)	35	35	NR	
	Kvien 2008 [21]*	AUSCAN	0-100	-8.1 (17.5)	-3.6 (17.3)	42	41	-4.5 (-12.2 to -3.2)	
Intra-articular	Sabaah 2020(a) [31]	AUSCAN	0-20	16.7 (4.9)	16 (3.3)	15	15	NR	-0.55 (-1.19, 0.09)
	Sabaah 2020(b) [31]	AUSCAN	0-20	16.7 (4.9)	19.3 (1.6)	15	15	NR	
	Bahadir 2009 [27]	Duruoz hand index	0-90	13.8 (10.2)	24 (12.4)	20	20	NR	
Intermediate term effect (3 months/12-14 weeks)/pain									
Oral	Kroon 2019 [8]*	VAS	0-100	NA	NA	46	46	6.6 (-3.7 to 16.9)	MD 4.06 (-1.53, 9.65)
	Wenham 2012 [22]*	VAS	0-100	-10 (13.9)	-13 (13.9)	34	33	NR	
Intra-articular	Malahias 2021 [32]	VAS	0-100	30.83 (42.44)	42.5 (42.67)	17	16	NR	0.35 (-0.63, 1.33)
	Sabaah 2020(a) [31]	VAS	0-10	6 (2.5)	2.7 (0.8)	15	15	NR	
	Sabaah 2020(b) [31]	VAS	0-10	6 (2.5)	5 (1.6)	15	15	NR	
	Bahadir 2009 [27]	VAS	0-100	32 (20)	46 (27)	20	20	NR	
Intermediate term effect (3 months)/function									
Oral	Kroon 2019 [8]*	AUSCAN	0-20	-1.3 (6.8)	-1.8 (6.3)	46	46	0.7 (-2 to 3.4)	-0.04 (-0.35, 0.27)
	Wenham 2012 [22]*	AUSCAN	0-100	-2 (10.1)	0 (10.1)	34	33	NR	
Intra-articular	Malahias 2021 [32]	Q-DASH	0-100	32.6 (31.8)	32.8 (29.2)	17	16	NR	0.39 (-0.79, 1.56)
	Sabaah 2020(a) [31]	AUSCAN	0-20	21.7 (2.5)	14 (3.3)	15	15	NR	
	Sabaah 2020(b) [31]	AUSCAN	0-20	21.7 (2.5)	20.3 (3.3)	15	15	NR	
	Bahadir 2009 [27]	Duruoz hand index	0-90	11.2 (8.5)	22.2 (13.2)	20	20	NR	
Long term effect (6 months)/pain									
Intra-articular	Jahangiri 2014 [28]	VAS	0-10	2.4 (1.8)	1.2 (1.6)	27	28	NR	-0.18 (-1.91, 1.55)
	Bahadir 2009 [27]	VAS	0-10	3.5 (1.8)	5.7 (2.2)	20	20	NR	
Long term effect (6 months)/function									
Intra-articular	Jahangiri 2014 [28]	HAQ-DI	0-3	2.6 (1.5)	1.6 (1.3)	27	28	NR	-0.10 (-1.69, 1.49)
	Bahadir 2009 [27]	Duruoz Hand Index	0-90	12 (8.7)	20 (22.1)	20	20	NR	
Long term effect (12 months)/pain									
Intra-articular	Malahias 2021 [32]	VAS	0-10	65 (24.3)	27.5 (34.5)	17	16	NR	0.34 (-1.38, 2.06)
	Bahadir 2009 [27]	VAS	0-10	3.5 (1.8)	5.7 (2.2)	20	20	NR	
Long term effect (12 months)/function									
Intra-articular	Malahias 2021 [32]	Q-DASH	0-100	43 (27.6)	20.4 (27.7)	17	16	NR	0.24 (-0.84, 1.31)
	Bahadir 2009 [27]	Duruoz Hand Index	0-90	21.1 (11.6)	24.9 (13.4)	20	20	NR	

*mean change from baseline

Sabaah 2020(a): corticosteroid vs hyaluronic acid, Sabaah 2020 b) corticosteroid vs platelet-rich plasma

*AUSCAN* Australian Canadian Osteoarthritis Hand Index, *CI* Confidence interval, *Q-DASH* Quick Disabilities of the Arm, Shoulder and Hand questionnaire, *HAQ-DI* Health Assessment Questionnaire Disability Index Questionnaire, *MD* Mean difference, *NR* Not reported, *SD* Standard deviation, *SMD* Standardized mean difference, *VAS* Visual analogue scale

**Fig. 3:**
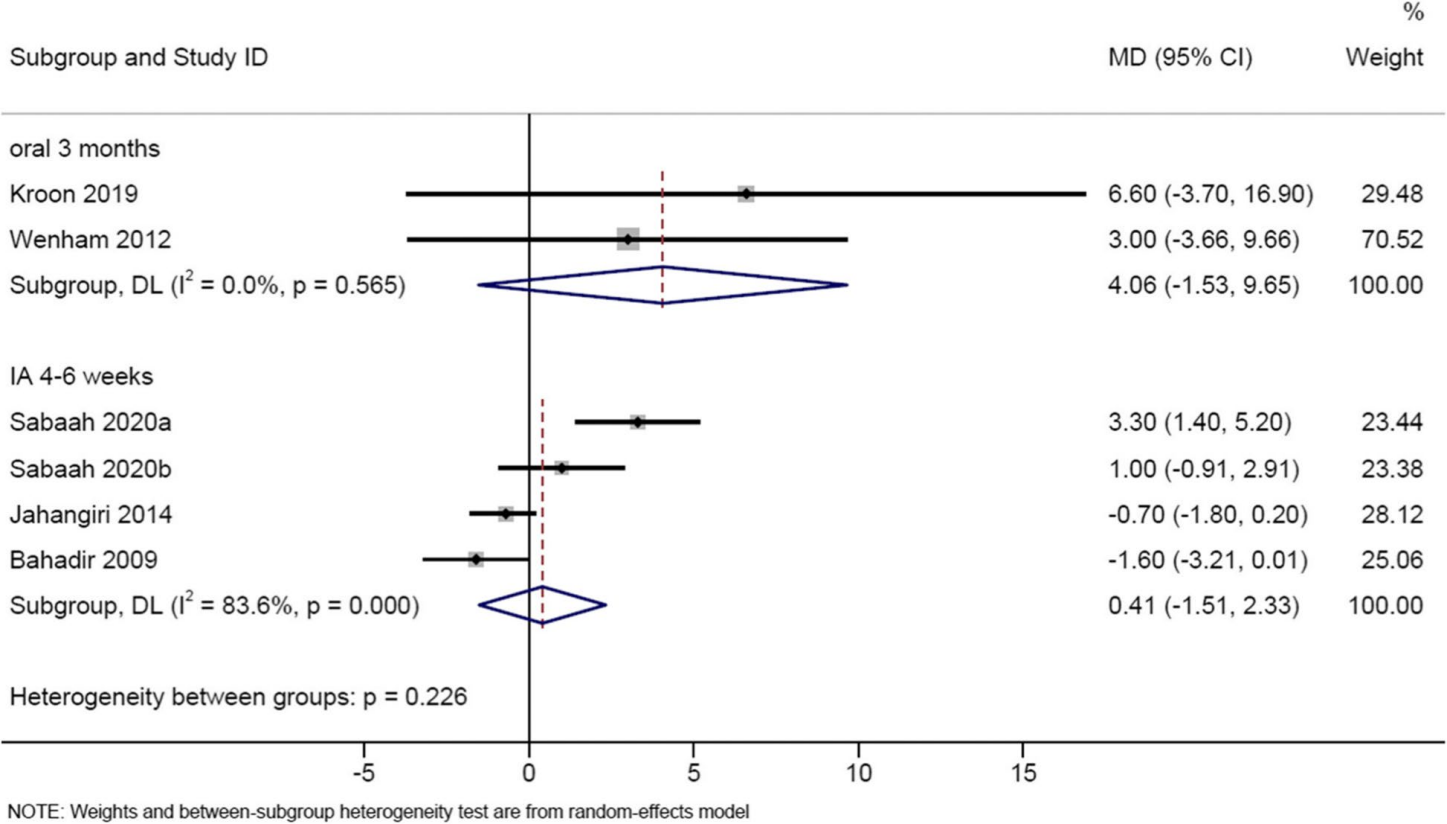
Random-effects meta-analysis of the mean difference in pain, corticosteroid vs control on treatment (4-6 weeks) and off treatment follow-up (3-12 months)

Ten studies evaluated intra-articular corticosteroids compared to placebo in thumb carpometacarpal OA [23–29, 31, 32] or interphalangeal OA [30] (Table 2; Supplementary Table 3). Due to data unavailability and high heterogeneity, meta-analysis was performed on two studies [27, 31] for SMD (-0.29, 95% CI -0.74 to 0.15) (Fig. 2), and three studies [27, 28, 31] for MD (0.41, 95% CI -1.51 to 2.33; using visual analogue scale, VAS) (Fig. 3), showing no significant effect on pain in thumb carpometacarpal OA. Meta-analysis of two studies [27, 31] showed no beneficial effect of corticosteroids on pain vs hyaluronic acid or platelet-rich plasma (MD 0.86, 95% CI -2.00 to 3.72, using VAS). Fuchs et al showed significantly faster pain reduction by 2-3 weeks from corticosteroid injection compared to sodium hyaluronate [25]. Other studies did not show significant difference between corticosteroid and control groups [23, 24, 26, 28, 29, 32]. Spolidoro et al showed a greater improvement in pain at movement from corticosteroid injection compared to placebo in interphalangeal OA at 4 weeks [30].

### Short-term effect of corticosteroids on function

**Fig. 4:**
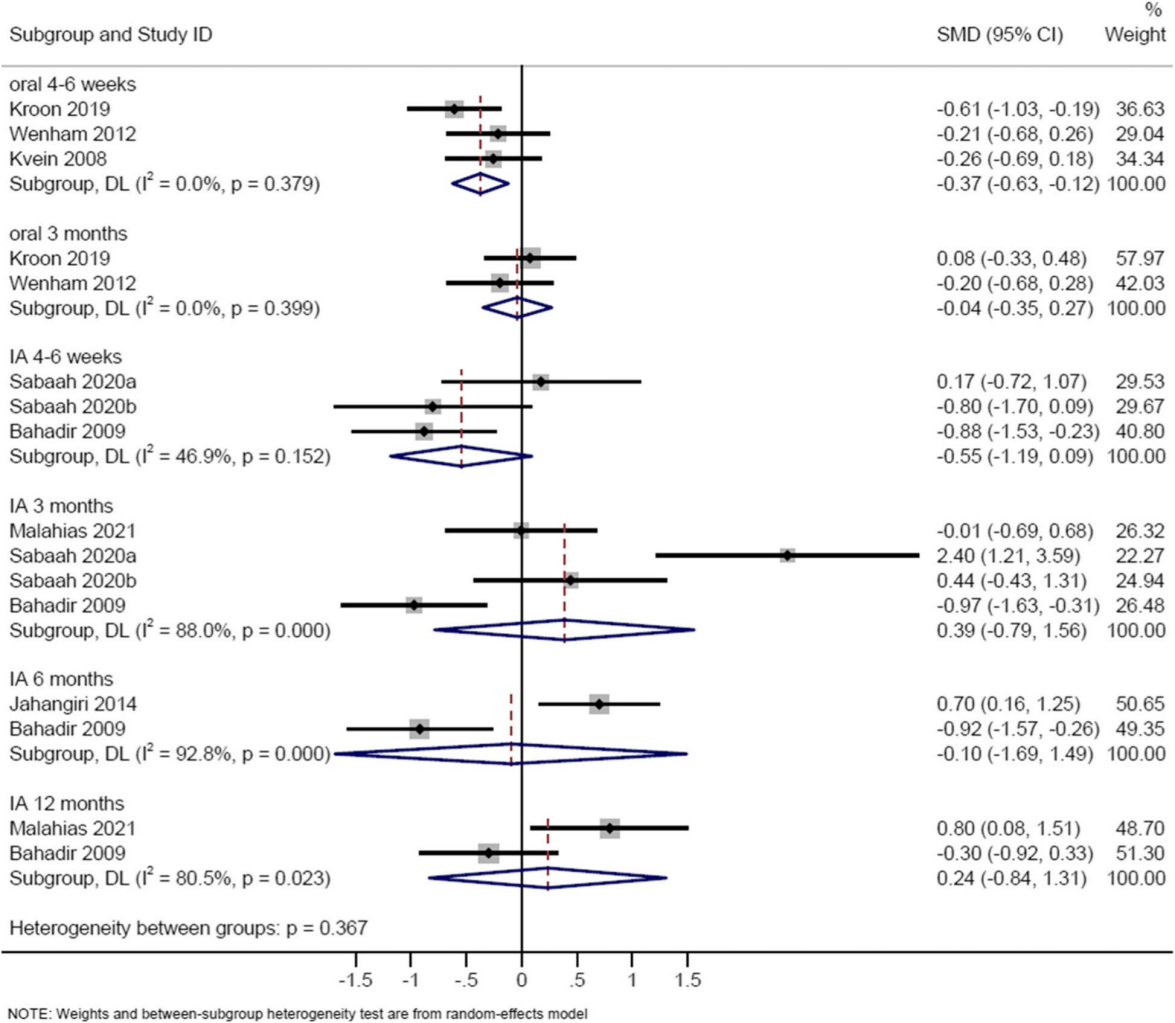
Random-effects meta-analysis of the standard mean difference in function, corticosteroid vs control on treatment (4-6 weeks) and off treatment follow-up (3-12 months)

Three studies evaluated oral corticosteroids compared to placebo [8, 21, 22] (Table 2; Supplementary Table 2), with meta-analysis showing favourable effect of corticosteroids on function (SMD -0.37, 95% CI -0.63 to -0.12) (Fig. 4). The result was similar when Kvien’s study [21] was excluded (SMD -0.43, 95% CI -0.81 to -0.04).

Six studies evaluated intra-articular corticosteroids compared to placebo [26–31] (Table 2; Supplementary Table 3). Meta-analysis of two studies [27, 31] showed no significant effect of corticosteroids on function in thumb carpometacarpal OA (SMD -0.55, 95% CI -1.19 to 0.09) (Fig. 4). Jahangiri showed corticosteroids improved function at 2 months but not at 1 month [28]. Other studies demonstrated no significant effect on function in thumb carpometacarpal OA [26, 29] or interphalangeal OA [30].

### Short-term effect of corticosteroids on other outcomes

Two studies examined oral corticosteroids on stiffness [21, 22], with meta-analysis showing favourable effect on improving stiffness (MD -5.03, 95% CI -9.91 to -0.15; using Australian Canadian Osteoarthritis Hand Index (AUSCAN) stiffness subscale) (Supplementary Figure 2). Kroon et al found oral corticosteroid increased the fulfilment of OMERAT-OARSI responder criteria, with no effect on grip strength [8] (Supplementary Table 2). This study also showed oral corticosteroids reduced the summed score of synovial thickening by ultrasound and bone marrow lesions by MRI at 6 weeks, with no effect on synovitis by MRI or power doppler signal [8]. Wenham et al reported no effect on synovitis by MRI at 4 weeks [22] (Supplementary Table 2).

Intra-articular corticosteroids showed no beneficial effect on grip strength [24, 26, 27, 30, 31], lateral pinch [24, 26, 27], tip pinch [26, 27], pinch strength [28, 30, 31], chunk pinch [27], pain intensity on pressure [28], pain threshold [28], tenderness [23], palpation for joint tenderness [31], Provocative tests [31], or swollen joint count [30] (Supplementary Table 3).

### Intermediate-term (12-14 weeks/3 months) effect of corticosteroids on pain

Two studies evaluated the posttreatment effect of oral corticosteroids compared to placebo [8, 22] at 12-14 weeks which was 6-8 weeks after study medication was stopped (Table 2; Supplementary Table 2), with meta-analysis showing no effect on pain (MD 4.06, 95% CI -1.53 to 9.65; using VAS) (Fig. 3).

Ten studies evaluated intra-articular corticosteroids compared to placebo [23–32] (Table 2; Supplementary Table 3). Meta-analysis of three studies [27, 31, 32] showed no effect on pain in thumb carpometacarpal OA (SMD 0.35, 95% CI -0.63 to 1.33) (Fig. 2). Other studies found no effect of corticosteroids in thumb carpometacarpal OA [23–26, 28, 29]. Spolidoro et al showed corticosteroid injection resulted in a greater improvement in pain at movement compared to placebo in interphalangeal OA at 12 weeks [30].

### Intermediate-term effect of corticosteroids on function

Two studies evaluated oral corticosteroids compared to placebo [8, 22] (Table 2; Supplementary Table 2), with meta-analysis demonstrating no effect on function (SMD -0.04, 95% CI -0.35 to 0.27) (Fig. 4).

Six studies evaluated intra-articular corticosteroids compared to placebo [23, 26, 27, 30–32] (Table 2; Supplementary Table 3). Meta-analysis of three studies [27, 31, 32] showed no effect on function in thumb carpometacarpal OA (SMD 0.39, 95% CI -0.79 to 1.56) (Fig. 4). Other studies demonstrated no effect of corticosteroids in thumb carpometacarpal OA [23, 26] or interphalangeal OA [30].

### Intermediate-term effect of corticosteroids on other outcomes

Oral or intra-articular corticosteroids showed no significant effect on stiffness [8, 22], grip strength [24, 26, 27, 30], lateral pinch [24, 26, 27], tip pinch [26, 27], pinch strength [30], chunk pinch [27], tenderness [23], swollen joint count [30], fulfilment of OMERACT-OARSI responder criteria [8], palpation for joint tenderness [31], Provocative tests [31], or patient satisfaction [32] (Supplementary Table 3).

### Long-term (6-12 months) effect of corticosteroids on pain

For intra-articular corticosteroids, seven studies examined pain at 6 months [23–29] and two studies at 12 months [27, 32] in thumb carpometacarpal OA (Table 2; Supplementary Table 3). Meta-analysis of two studies showed no effect of intra-articular corticosteroids on pain at 6 months (SMD -0.18, 95% CI -1.91 to 1.55) [23, 30] and 12 months (SMD 0.34, 95% CI -1.38 to 2.06) [30, 31] (Fig. 2). Other studies found no favourable effect on pain [23–26, 29].

### Long-term effect of corticosteroids on function

For intra-articular corticosteroids, four studies examined function at 6 months [26–29] and two studies at 12 months [27, 32] in thumb carpometacarpal OA (Table 2; Supplementary Table 3). Meta-analysis of two studies showed no effect of intra-articular corticosteroids on function at 6 months (SMD -0.10, 95% CI -1.69 to 1.49) [23, 30] and 12 months (SMD 0.24, 95% CI -0.84 to 1.31) [30, 31] (Fig. 4). Other studies found no effect on function at 6 months [26, 29].

### Long-term effect of corticosteroids on other outcomes

Intra-articular corticosteroids showed no significant effect on grip strength [24, 26, 27], lateral pinch [24, 26, 27], tip pinch [26, 27], pinch strength [28], chunk pinch [27], pain intensity on pressure [28], pain threshold [28], tenderness [23], or patient satisfaction [32] in thumb carpometacarpal OA (Supplementary Table 3).

### Assessment of risk of bias due to missing evidence and certainty in the body of evidence

There was some concern about risk of bias due to missing evidence in meta-analyses of pain and function in comparison to control (Supplementary Tables 5 and 6). The quality of evidence from our meta-analysis was low (Supplementary Tables 7 and 8). We downgraded the evidence because of risk of bias in studies, heterogeneity, and imprecision.

## Discussion

Our systematic review and meta-analysis demonstrated that oral corticosteroids had a medium effect on reducing pain and stiffness and small-to-medium effect on improving function over 4-6 weeks while on treatment, but the effect did not persist over longer term (3 months) which was 6-8 weeks after treatment was ended. Intra-articular corticosteroids showed no significant effect on any clinical outcomes over short (4-6 weeks) or longer term (3-12 months) in thumb carpometacarpal OA. Two trials evaluated joint structure at 4-6 weeks with one study showing oral corticosteroids reduced synovial thickening on ultrasound but neither showed an effect on synovitis assessed by MRI. None of the studies examined the effect of corticosteroids on structural outcomes over longer term.

No previous systematic review has examined the efficacy of oral corticosteroids on both clinical and structural outcomes in hand OA, based on the duration of treatment effect (short term vs. longer term). Our meta-analysis of three studies [8, 21, 22] (two with low risk of bias and one with high risk of bias) showed a clinically significant benefit of oral corticosteroids for pain control and functional improvement in hand OA over 4-6 weeks, but the beneficial effect did not persist over longer term off treatment (i.e. at 3 months, which was 6-8 weeks after the medication was stopped). There were no clinical trials examining the effect of oral corticosteroids on disease progression of hand OA. Two studies examined the effect of oral corticosteroids on joint structures with inconclusive results [8, 22]. One study found oral corticosteroid reduced synovial thickening by ultrasound and bone marrow lesions by MRI at 6 weeks [8], neither study showed an effect on synovitis by MRI at 4-6 weeks [8, 22]. The effect of corticosteroids on disease progression warrants further investigations.

Our meta-analysis found no effect of intra-articular corticosteroids on pain control or functional improvement in thumb carpometacarpal OA at 4-6 weeks. This contrasts with the findings at other joints where intra-articular corticosteroids reduced pain and improved function in knee OA over 4-6 weeks [33] and reduce pain in hip OA over 3-4 weeks and 8-12 weeks [34]. There are a number of potential explanations for the effect of oral but not intra-articular corticosteroids on short term pain. In contrast to the studies of oral corticosteroids, there was significant heterogeneity in the drug, duration and dosage of corticosteroids in the 9 intra-articular studies with different doses of triamcinolone, methylprednisolone, or betamethasone used, with 4 studies using a single injection, 4 studies using weekly injection over 2-3 weeks, and one study using monthly injection for 3 months. The oral corticosteroid studies examined patients who had predominantly interphalangeal OA, but 8 of 9 intra-articular corticosteroid studies examined patients with carpometacarpal OA, which may have a different response to corticosteroids. Furthermore, intra-articular corticosteroid studies tended to have an active placebo where 6 studies used hyaluronic acid, one study used platelet-rich plasma, one study used lidocaine, and 2 studies used dextrose or saline, but all 3 oral corticosteroid studies used inactive placebo in the control group. Consistent with our findings, a previous systematic review on hand OA found no beneficial effect of intra-articular corticosteroids on pain and function at 26 weeks compared to placebo or hyaluronic acid in carpometacarpal OA [9]. As with our finding at the hand, intra-articular corticosteroids showed no effect on pain and function in a previous systematic review of OA at other joint sites over 12 months [35] and in clinical trials of knee OA over 1-2 years [36, 37]. There was only one trial comparing intra-articular corticosteroids with placebo in interphalangeal OA, showing a significant improvement in pain at movement at 1, 4, 8, and 12 weeks [30]. The effect of intra-articular corticosteroids in interphalangeal OA requires further investigation.

Currently clinical guidelines for the management of hand OA do not strongly recommend the use of corticosteroids [6, 7]. Our findings suggest that oral corticosteroids could be used for improving pain and function in hand OA over 4-6 weeks. However, the use of oral corticosteroids will need to be carefully balanced against the potential for significant adverse effects, especially in the absence of a disease-modifying agent and the potential for ongoing and repeated use [38]. Only one study examined selected people with hand OA and evidence of synovitis [8]. Hand OA is a heterogeneous disease with approximately 50% of those with symptomatic hand OA having evidence of synovitis [39, 40] which causes pain and disease progression [39, 41]. Further work is needed to determine whether there are some patients with hand OA in whom the benefits of oral corticosteroids outweigh the risks. The potential benefit would be strengthened if there was evidence of decreased synovitis and the potential of reduced joint damage. However, no study has shown this although one study found reduced synovial thickening and bone marrow lesions at 6 weeks of oral corticosteroid [8]. Any use of oral corticosteroids would need clear guidelines as to duration of treatment and criteria for cessation.

This is the first systematic review and meta-analysis to comprehensively evaluate the available data on efficacy of oral and intra-articular corticosteroids on symptoms and structural outcomes in hand OA. Our systematic review included 13 trials with broad examination of outcomes, in contrast to a recently published systematic review and meta-analysis which only included 7 trials with a focus on pain lasting for up to 24 weeks and safety [12]. We wanted to see whether there were any dimensions where corticosteroids might be effective. Our systematic review was performed in accordance with the PRISMA guideline, with a comprehensive search performed in three databases in addition to clinical trial registries to identify unpublished trials. The RoB 2 tool was used to assess risk of bias and the recently developed ROB-ME tool to evaluate risk of bias due to missing evidence. Our study has limitations. There was heterogeneity in terms of study population, formulation and dosage of corticosteroids, protocol and duration of treatment, comparator, outcome measures, and length of follow-up, therefore different treatment effects may have arisen. We were unable to perform meta-analysis for most of the intra-articular studies due to lack of usable data and heterogeneity of studies. Most of the studies had some concerns or high risk of bias which was congruent with the GRADE and ROB-ME assessments. Thus, the certainty of the evidence for the efficacy of corticosteroids in improving pain and function in hand OA is low. As most of the studies had moderate sample size, the 95% CI of treatment effect was wide even after combining the results with low effect size. These reduce our ability to demonstrate a clinically meaningful effect and the results need to be viewed with caution.

## Conclusions

There was low-certainty evidence for an effect of oral corticosteroids on improving pain, stiffness and function in hand OA over 4-6 weeks, with no significant effect persisting off treatment over longer term (3 months). Care is needed in interpreting the results of oral corticosteroids given the potential for harm especially with no evidence to date of a disease-modifying effect. Intra-articular corticosteroids had no significant effect on clinical outcomes in carpometacarpal OA, with one trial showing an effect of intra-articular corticosteroids on improving pain during movement in interphalangeal OA. More work is needed to clarify the role of corticosteroids, oral or intra-articular, in the management of hand OA.

## Supplementary Information

**Additional file 1: Supplementary Table 1.** Search strategy for systematic review in Ovid MEDLINE(R) and Epub Ahead of Print, In-Process, In-Data-Review & Other Non-Indexed Citations, Daily and Versions(R)/Embase/Cochrane, Ovid Embase Classic+Embase, Ovid EBM Reviews - Cochrane Central Register of Controlled Trials. **Supplementary Table 2.** Overview of raw data for studies evaluating oral corticosteroids for all outcomes at all time points. **Supplementary Table 3.** Overview of raw data for studies evaluating intra-articular corticosteroids for all outcomes at all time points. **Supplementary Table 4.** Search of clinical trial registers and registries for trials with Completed or Unknown status that are not published. **Supplementary Table 5.** Rob Me assessment for random effect meta-analysis of the effect of oral corticosteroid vs placebo on pain at short term (4-6 weeks). **Supplementary Table 6.** Rob Me assessment for random effect meta-analysis of the effect of intra-articular corticosteroid vs placebo on pain at short term (4-6 weeks). **Supplementary Table 7.** GRADE assessment for random effect meta-analysis of the effect of oral corticosteroid vs placebo on pain at short term (4-6 weeks). **Supplementary Table 8.** GRADE assessment for random effect meta-analysis of the effect of intra-articular corticosteroid vs placebo on pain at short term (4-6 weeks). **Supplementary Figure 1.** Risk of bias assessment using RoB 2 tool considering patient reported pain and functional outcome. **Supplementary Figure 2.** Random effects meta-analysis of the standard mean difference in stiffness, based on oral corticosteroid or placebo at 4-6 weeks.

## Data Availability

The datasets generated and/or analysed during the current study are available in the Open Science Framework, https://osf.io/dk283/.

## Abbreviations

ACR: American College of Rheumatology
AUSCAN: Australian Canadian Osteoarthritis Hand Index
CI: Confidence interval
CMC: Carpometacarpal joint
DIP: Distal interphalangeal joint
EL: Eaton and Litter classification FIHOA Functional Index for Hand Osteoarthritis
HA: Hyaluronic acid
HAQ-DI: Health Assessment Questionnaire Disability Index Questionnaire
IP: Interphalangeal joint
KL: Kellgren-Lawrence
MD: Mean difference
MRI: Magnetic resonance imaging
NSAIDs: Non-steroidal anti-inflammatory drugs
OA: Osteoarthritis
OARSI: Osteoarthritis Research Society International
OMERACT: Outcome Measures in Rheumatology
PIP: Proximal interphalangeal joint
PRISMA: Preferred Reporting Items for Systematic Review and Meta-Analysis
PRP: Platelet-rich plasma
Q-DASH: Quick Disabilities of the Arm, Shoulder and Hand questionnaire
RoB: Risk of bias
SD: Standard deviation
SMD: Standardized mean difference
TMJ: Trapeziometacarpal joint
VAS: Visual analogue scale

## References

[1] Zhang Y, Niu J, Kelly-Hayes M, Chaisson CE, Aliabadi P, Felson DT (2002). Prevalence of Symptomatic Hand Osteoarthritis and Its Impact on Functional Status among the Elderly: The Framingham Study. Am J Epidemiol.

[2] Kjeken I, Dagfinrud H, Slatkowsky-Christensen B, Mowinckel P, Uhlig T, Kvien TK, Finset A (2005). Activity limitations and participation restrictions in women with hand osteoarthritis: patients' descriptions and associations between dimensions of functioning. Ann Rheum Dis.

[3] Loef M, Damman W, de Mutsert R, Rosendaal FR, Kloppenburg M (2020). Health-related Quality of Life in Patients with Hand Osteoarthritis from the General Population and the Outpatient Clinic. J Rheumatol.

[4] Dillon CF, Hirsch R, Rasch EK, Gu Q (2007). Symptomatic hand osteoarthritis in the United States: prevalence and functional impairment estimates from the third U.S. National Health and Nutrition Examination Survey, 1991-1994. Am J Phys Med Rehabil.

[5] Dahaghin S, Bierma-Zeinstra SM, Ginai AZ, Pols HA, Hazes JM, Koes BW (2005). Prevalence and pattern of radiographic hand osteoarthritis and association with pain and disability (the Rotterdam study). Ann Rheum Dis.

[6] Kloppenburg M, Kroon FPB, Blanco FJ, Doherty M, Dziedzic KS, Greibrokk E, Haugen IK, Herrero-Beaumont G, Jonsson H, Kjeken I (2019). 2018 update of the EULAR recommendations for the management of hand osteoarthritis. Ann Rheum Dis.

[7] Kolasinski SL, Neogi T, Hochberg MC, Oatis C, Guyatt G, Block J, Callahan L, Copenhaver C, Dodge C, Felson D (2020). 2019 American College of Rheumatology/Arthritis Foundation Guideline for the Management of Osteoarthritis of the Hand, Hip, and Knee. Arthritis Care Res.

[8] Kroon FPB, Kortekaas MC, Boonen A, Böhringer S, Reijnierse M, Rosendaal FR, Riyazi N, Starmans M, Turkstra F, van Zeben J (2019). Results of a 6-week treatment with 10 mg prednisolone in patients with hand osteoarthritis (HOPE): a double-blind, randomised, placebo-controlled trial. Lancet.

[9] Kroon FPB, Rubio R, Schoones JW, Kloppenburg M (2016). Intra-Articular Therapies in the Treatment of Hand Osteoarthritis: A Systematic Literature Review. Drugs Aging.

[10] Lue S, Koppikar S, Shaikh K, Mahendira D, Towheed TE (2017). Systematic review of non-surgical therapies for osteoarthritis of the hand: an update. Osteoarthr Cartil.

[11] Kroon FPB, Carmona L, Schoones JW, Kloppenburg M (2018). Efficacy and safety of non-pharmacological, pharmacological and surgical treatment for hand osteoarthritis: a systematic literature review informing the 2018 update of the EULAR recommendations for the management of hand osteoarthritis. RMD Open.

[12] Wang X, Wang P, Faramand A, Zha X, Zhang Y, Chong W, et al. Efficacy and safety of corticosteroid in the treatment of hand osteoarthritis: a systematic review and meta-analysis of randomized controlled trials. Clin Rheumatol. 2022. 10.1007/s10067-021-06024-8 35091776

[13] Page MJ, McKenzie JE, Bossuyt PM, Boutron I, Hoffmann TC, Mulrow CD, Shamseer L, Tetzlaff JM, Akl EA, Brennan SE (2021). The PRISMA 2020 statement: an updated guideline for reporting systematic reviews. BMJ.

[14] Altman R, Alarcón G, Appelrouth D, Bloch D, Borenstein D, Brandt K, Brown C, Cooke TD, Daniel W, Gray R (1990). The American College of Rheumatology criteria for the classification and reporting of osteoarthritis of the hand. Arthritis Rheum.

[15] Sterne JAC, Savović J, Page MJ, Elbers RG, Blencowe NS, Boutron I, Cates CJ, Cheng H-Y, Corbett MS, Eldridge SM (2019). RoB 2: a revised tool for assessing risk of bias in randomised trials. BMJ.

[16] McGuinness LA, Higgins JPT (2021). Risk-of-bias VISualization (robvis): An R package and Shiny web app for visualizing risk-of-bias assessments. Res Synth Methods.

[17] Higgins JPTTJ, Chandler J, Cumpston M, Li T, Page MJ, Welch VA (2019). (editors): Cochrane Handbook for Systematic Reviews of Interventions.

[18] DerSimonian R, Laird N (1986). Meta-analysis in clinical trials. Control Clin Trials.

[19] Higgins JPT, Thompson SG, Deeks JJ, Altman DG (2003). Measuring inconsistency in meta-analyses. BMJ.

[20] Hultcrantz M, Rind D, Akl EA, Treweek S, Mustafa RA, Iorio A, Alper BS, Meerpohl JJ, Murad MH, Ansari MT (2017). The GRADE Working Group clarifies the construct of certainty of evidence. J Clin Epidemiol.

[21] Kvien TK, Fjeld E, Slatkowsky-Christensen B, Nichols M, Zhang Y, Prøven A, Mikkelsen K, Palm Ø, Borisy AA, Lessem J (2008). Efficacy and safety of a novel synergistic drug candidate, CRx-102, in hand osteoarthritis. Ann Rheum Dis.

[22] Wenham CYJ, Hensor EMA, Grainger AJ, Hodgson R, Balamoody S, Dore CJ, Emery P, Conaghan PG (2012). A randomized, double-blind, placebo-controlled trial of low-dose oral prednisolone for treating painful hand osteoarthritis. Rheumatology (Oxford).

[23] Meenagh GK, Patton J, Kynes C, Wright GD (2004). A randomised controlled trial of intra-articular corticosteroid injection of the carpometacarpal joint of the thumb in osteoarthritis. Ann Rheum Dis.

[24] Stahl S, Karsh-Zafrir I, Ratzon N, Rosenberg N (2005). Comparison of Intraarticular Injection of Depot Corticosteroid and Hyaluronic Acid for Treatment of Degenerative Trapeziometacarpal Joints. J Clin Rheumatol.

[25] Fuchs S, Mönikes R, Wohlmeiner A, Heyse T (2006). Intra-articular hyaluronic acid compared with corticoid injections for the treatment of rhizarthrosis. Osteoarthr Cartil.

[26] Heyworth BEMD, Lee JHMD, Kim PDMD, Lipton CBMD, Strauch RJMD, Rosenwasser MPMD (2008). Hylan Versus Corticosteroid Versus Placebo for Treatment of Basal Joint Arthritis: A Prospective, Randomized, Double-Blinded Clinical Trial. J Hand Surg [Am].

[27] Bahadır C, Onal B, Dayan VY, Gürer N (2009). Comparison of therapeutic effects of sodium hyaluronate and corticosteroid injections on trapeziometacarpal joint osteoarthritis. Clin Rheumatol.

[28] Jahangiri A, Moghaddam FR, Najafi S (2014). Hypertonic dextrose versus corticosteroid local injection for the treatment of osteoarthritis in the first carpometacarpal joint: a double-blind randomized clinical trial. J Orthop Sci.

[29] Monfort J, Rotés-Sala D, Segalés N, Montañes F-J, Orellana C, Llorente-Onaindia J, Mojal S, Padró I, Benito P (2014). Comparative efficacy of intra-articular hyaluronic acid and corticoid injections in osteoarthritis of the first carpometacarpal joint: Results of a 6-month single-masked randomized study. Joint Bone Spine.

[30] Spolidoro Paschoal Nde O, Natour J, Machado FS, de Oliveira HA, Furtado RN (2015). Effectiveness of Triamcinolone Hexacetonide Intraarticular Injection in Interphalangeal Joints: A 12-week Randomized Controlled Trial in Patients with Hand Osteoarthritis. J Rheumatol.

[31] Abdelsabor Sabaah HM, El Fattah RA, Al Zifzaf D, Saad H (2020). A Comparative Study for Different Types of Thumb Base Osteoarthritis Injections: A Randomized Controlled Interventional Study. Ortop Traumatol Rehabil.

[32] Malahias M-A, Roumeliotis L, Nikolaou VS, Chronopoulos E, Sourlas I, Babis GC (2021). Platelet-Rich Plasma versus Corticosteroid Intra-Articular Injections for the Treatment of Trapeziometacarpal Arthritis: A Prospective Randomized Controlled Clinical Trial. Cartilage.

[33] Jüni P, Hari R, Rutjes AWS, Fischer R, Silletta MG, Reichenbach S, et al. Intra-articular corticosteroid for knee osteoarthritis. Cochrane Database Syst Rev. 2015:CD005328. 10.1002/14651858.CD005328.pub3 PMC8884338 26490760

[34] Zhong H-M, Zhao G-F, Lin T, Zhang X-X, Li X-Y, Lin J-F, Zhao S-Q, Pan Z-J (2020). Intra-Articular Steroid Injection for Patients with Hip Osteoarthritis: A Systematic Review and Meta-Analysis. Biomed Res Int.

[35] Ayub S, Kaur J, Hui M, Espahbodi S, Hall M, Doherty M, Zhang W (2021). Efficacy and safety of multiple intra-articular corticosteroid injections for osteoarthritis-a systematic review and meta-analysis of randomized controlled trials and observational studies. Rheumatology.

[36] Raynauld JP, Buckland-Wright C, Ward R, Choquette D, Haraoui B, Martel-Pelletier J, Uthman I, Khy V, Tremblay JL, Bertrand C, Pelletier JP (2003). Safety and efficacy of long-term intraarticular steroid injections in osteoarthritis of the knee: a randomized, double-blind, placebo-controlled trial. Arthritis Rheum.

[37] McAlindon TE, LaValley MP, Harvey WF, Price LL, Driban JB, Zhang M, Ward RJ (2017). Effect of Intra-articular Triamcinolone vs Saline on Knee Cartilage Volume and Pain in Patients With Knee Osteoarthritis: A Randomized Clinical Trial. JAMA.

[38] Manson SC, Brown RE, Cerulli A, Vidaurre CF (2009). The cumulative burden of oral corticosteroid side effects and the economic implications of steroid use. Respir Med.

[39] Keen HI, Wakefield RJ, Grainger AJ, Hensor EM, Emery P, Conaghan PG (2008). An ultrasonographic study of osteoarthritis of the hand: synovitis and its relationship to structural pathology and symptoms. Arthritis Rheum.

[40] Kortekaas MC, Kwok WY, Reijnierse M, Watt I, Huizinga TW, Kloppenburg M (2010). Pain in hand osteoarthritis is associated with inflammation: the value of ultrasound. Ann Rheum Dis.

[41] Keen HI, Lavie F, Wakefield RJ, D'Agostino MA, Hammer HB, Hensor E, Pendleton A, Kane D, Guerini H, Schueller-Weidekamm C (2008). The development of a preliminary ultrasonographic scoring system for features of hand osteoarthritis. Ann Rheum Dis.

