# Supplementary material for: Efficacy of corticosteroids for hand osteoarthritis - a systematic review and meta-analysis of randomized controlled trials
Source: BMC Musculoskelet Disord. 2022 Jul 13;23:665. doi: 10.1186/s12891-022-05619-9 (PMC9277790; doi:10.1186/s12891-022-05619-9)

Supplementary Table 1. Search strategy for systematic review in Ovid MEDLINE(R) and Epub Ahead of Print, In-Process, In-Data-Review & Other Non-Indexed Citations, Daily and Versions(R)/Embase/Cochrane, Ovid Embase Classic+Embase, Ovid EBM Reviews - Cochrane Central Register of Controlled Trials

	Search term/ key words
1	(hand* or "hand joint*" or "ïntermetacarpal joint*" or finger* or "finger joint*" or "carpal joint*" or digit* or "carpometacarpal joint*" or "metacarpophalangeal joint" or thumb* or metacarpus or "Thumb Carpometacarpal Joint" or "first metacarpal-carpal" or carpometacarpal or interphalangeal or "distal interphalangeal" or "proximal interphalangeal" or intermetacarp* or interphalang* or intercarp* or carpometacarp* or metacarpophalang* or metacarp* or CMC or IP or "thumb base*" or TB).mp.
2	(Osteoarthritis or Osteo-arthritis or osteoarthr* or osteo-arthr* or Osteo-arthritis or osteoarthr* or osteo-arthr* or osteoarthrosis or "degenerative arthritis" or "erosive osteoarthritis" or "arthrosis" or "arthroses" or "OA" or HOA).mp.
3	("randomized controlled trial" or "randomised controlled trial" or "controlled trial" or "multicentre randomised controlled trial" or "multicentre randomized controlled trial" or "randomized single-blind controlled trial" or "randomised single-blind controlled trial" or "Prospective, Randomized Study" or "Prospective, Randomised Study" or "double-blind, randomised, placebo-controlled trial" or "double-blind, randomized, placebo-controlled trial" or "randomised study" or "randomized study" or "Randomized Trial" or "Randomised Trial" or "randomized, single-blind, prospective study" or "randomised, single-blind, prospective study" or "randomised, double-blind, placebo-controlled trial" or "randomized, double-blind, placebo-controlled trial" or "randomized clinical trial" or "randomised clinical trial" or "clinical trial" or "randomised double-blind placebo-controlled crossover trial" or "randomized double-blind placebo-controlled crossover trial" or "randomised double-blind placebo-controlled trial" or "randomized double-blind placebo-controlled trial").mp.
4	(Steroids or corticosteroid* or prednisolone or methyl prednisolone or Methylprednisolone or Depo medrone or depomedrone or solu Medrol or Triamcinolone or triamcinolone acetonide or triamcinolone or Kenalog or betamethasone or celestone or celestone soluspan or glucocorticoid*).mp.
5	1 and 2 and 3 and 4
6	Limit to English language

Supplementary Table 2. Overview of raw data for studies evaluating oral corticosteroids for all outcomes at all time points

Study<br>Year<br>Country	Outcome	Outcome measures	End points	Intervention	Control	Mean difference (95% CI)/OR (95% CI)/RR (95% CI)	P value
Oral prednisolone vs placebo							
Kroon<br>(2019)<br>The<br>Netherla<br>nds	Change of pain score from baseline Baseline - 3 months (between group mean difference reduced at week 14)	VAS (0-100)	Baseline<br>6 weeks<br>8 weeks<br>14 weeks	-54.4 (21.8)<br>-21.5 (21.7)<br>- NR<br>- NR	-53.6 (19.3)<br>-5.2 (24.3)<br>- NR<br>- NR	-16.5 (-26.1 to -6.9)<br>-8.5 (-18.5 to 1.5)<br>6.6 (-3.7 to 16.9)	0.0007<br>-<br>-
		AUSCAN-Pain (0-20)	baseline<br>6 weeks<br>8 weeks<br>14 weeks	11.3 (3.3)<br>-4.7 (3.5)<br>-3.3 (3.5)<br>-0.4 (3.2)	10.2 (3.1)<br>-1.1 (3.1)<br>-1.4 (3.2)<br>-0.9 (3.7)	-3.5 (-4.9 to -2.1)<br>-1.7 (-3.2 to -0.3)<br>0.6 (-0.9 to 2.0)	<0.0001<br>0.02<br>0.43
		MHQ pain (0-100)	Baseline<br>6 weeks<br>8 weeks<br>14 weeks	50.9 (19)<br>-8.0 (17.9)<br>- NR<br>0.6 (12.9)	50.4 (14.4)<br>-5.0 (9.9)<br>- NR<br>-3.8 (13.6)	-3.7 (-11.7 to 4.4)<br>- NR<br>3.8 (-4.0 to 11.6)	0.37<br>-<br>0.34
	Fulfillment of outcome measures in OMERACT- OARSI responder criteria		6 weeks<br>14 weeks	-NR<br>-NR	-NR<br>-NR	5.3 (2.0 to 3.6)<br>0.9 (0.3 to 2.2)	0.0007<br>0.76
	Change of pain at thumb base	VAS	Baseline<br>6 weeks<br>8 weeks<br>14 weeks	34.3 (29)<br>-12·3 (28.0)<br>-7.9 (29.5)<br>4.3 (29.8)	35.4 (28.5)<br>0.2 (17.3)<br>0.4 (23.1)<br>3.6 (23.4)	-12.0 (-21.7 to -2.3)<br>-7.8 (-18.9 to 3.3)<br>1.3 (-9.9 to 12.6)	0.016<br>0.17<br>0.82
	Change of functional score from baseline	AUSCAN-Function	Baseline<br>6 weeks<br>8 weeks<br>14 weeks	18.6 (7.8)<br>-6.5 (7.4)<br>-4.5 (7.7)<br>-1.3 (6.8)	19.0 (7.1)<br>-2.7 (4.7)<br>-3.4 (5.4)<br>-1.8 (6.3)	-3.7 (-6.2 to -1.1)<br>-1.0 (-3.8 to 1.7)<br>0.7 (-2.0 to 3.4)	0.0051<br>0.46<br>0.49
		FIOHA	Baseline<br>6 weeks<br>8 weeks<br>14 weeks	12.4 (5.4)<br>-2.6 (5.1)<br>-1.3 (4.2)<br>0.6 (4.4)	11 (4.7)<br>-0.5 (4.0)<br>-0.8 (4.2)<br>0.5 (4.8)	-2.1 (-4.0 to -0.2)<br>-0.5 (-2.2 to 1.3)<br>0.1 (-1.8 to 2.1)	0.031<br>0.59<br>0.15
		MHQ overall function	Baseline<br>6 weeks<br>8 weeks<br>14 weeks	55.7 (13.9)<br>6.3 (13.2)<br>- NR<br>-4.2 (10.4)	57.1 (15.4)<br>0.2 (12.0)<br>- NR<br>-1.8 (12.0)	5.8 (0.5 to 11.1)<br>- NR<br>-2.5 (-7.2 to 2.3)	0.03<br>- NR<br>0.31
		MHQ activities of daily living	Baseline<br>6 weeks<br>8 weeks<br>14 weeks	70.2 (19.1)<br>8.2 (16.7)<br>- NR<br>-0.1 (13.3)	71.3 (16.7)<br>2.7 (12.9)<br>- NR<br>-0.2 (13.1)	5.5 (-0.8 to 11.7)<br>- NR<br>-0.3 (-6.0 to 5.4)	0.09<br>- NR<br>0.92
	Change in Grip strength from baseline		Baseline<br>6 weeks<br>8 weeks<br>14 weeks	20.4 (11.3)<br>3.5 (4.1)<br>- NR<br>-0.6 (5.2)	20.4 (11.8)<br>2.2 (5.5)<br>- NR<br>1.2 (6.1)	1.2 (-0.8 to 3.2)<br>- NR<br>-1.7 (-4.1 to 0.7)	0.24<br>- NR<br>0.16
	Synovial thickening (summed score)	Ultrasound (change from baseline)	Baseline<br>6 weeks<br>14 weeks	16.4 (6.3)<br>-2.8 (4.7)	17.8 (6.3)<br>-0.3 (5.0)	-2.5 (-4.5 to -0.5)	0.016
	Power Doppler signal	Ultrasound (change from baseline)	Baseline<br>6 weeks<br>14 weeks	5.3 (4.1)<br>-1.7 (4.3)	7.0 (4.3)<br>-1.3 (4.2)	-0.4 (-2.2 to 1.4)	0.68
	Synovitis	MRI (change from baseline)	Baseline<br>6 weeks<br>14 weeks	15.6 (7.5)<br>-0.7 (2.8)	14.8 (6.0)<br>-0.5 (2.2)	-0.2 (-1.4 to 0.9)	0.66
	Bone marrow lesions	MRI (change from baseline)	Baseline<br>6 weeks<br>14 weeks	11.0 (6.9)<br>-0.2 (1.7)	11.0 (6.7)<br>0.5 (1.2)	-0.7 (-1.3 to -0.02)	0.043
Kvien<br>*(*2008)<br>Norway	Pain	AUSCAN	Baseline<br>42 days (changes from baseline)	57.9 (20.2)<br>-14.2 (SE 3.0)	60.9 (19.4)<br>-4.0 (SE 3.1)	10.2 (1.6 to 18.7)	0.020
		VAS	Baseline	58.3 (20.1)	62.1 (16.9)		

			42 days (changes from baseline)	-18.6 (SE 3.3)	- 6.3 (3.3)	12.3 (3.0 to 21.5)	0.010
	Function	AUSCAN	Baseline<br>42 days (changes from baseline)	62.4 (19.5)<br>-8.1 (2.7)	67.8 (17.5)<br>-3.6 (2.7)	4.5 (-3.2 to 12.2)	0.246
	Stiffness	AUSCAN	Baseline<br>42 days (changes from baseline)	61.1 (18.0)<br>-15.2 (3.2)	64.5 (21.2)<br>-7.7 (3.3)	7.5 (-1.7 to 16.7)	0.108
Wenham (2012) UK	Pain over last 2 weeks (change from baseline)<br>Baseline - 3 months (change from baseline)	VAS	Baseline<br>Week 4<br>Week 12	62 (15)<br>-17 (-24, -11)<br>-8 (-16, 0)	58 (17)<br>-16 (-22, -9)<br>-8 (-16, 1)		0.77<br>0.94
	Pain over last 48 hours	VAS	Baseline<br>Week 4<br>Week 12	62 (19)<br>-20 (-27, -13)<br>-10 (-19, -1)	61 (16)<br>-17 (-24, -10)<br>-13 (-22, -4)		0.54<br>0.61
	Pain VAS worst joint last 48 hours (change from baseline)<br>Baseline - 3 months (change from baseline)	VAS	Baseline<br>Week 4<br>Week 12	71 (21)<br>-22 (-29, -15)<br>-10 (-19, 0)	67 (19)<br>-16 (-23, -9)<br>-8 (-17, 2)		0.19<br>0.78
	Pain	AUSCAN	Baseline<br>Week 4<br>Week 12	66 (16)<br>-18 (-24, -12)<br>-6 (-14, 1)	60 (15)<br>-12 (-18, -6)<br>-2 (-9, 6)		0.21<br>0.35
	Stiffness	AUSCAN	Baseline<br>Week 4<br>Week 12	55 (30)<br>-13 (-21, -5)<br>0 (-9, 9)	56 (21)<br>-9 (-17, -1)<br>0 (-9, 9)		0.46<br>0.95
	Function	AUSCAN	Baseline<br>Week 4<br>Week 12	61 (23)<br>-10 (-16, -4)<br>-2 (-8, 5)	62 (15)<br>-8 (-14, -2)<br>0 (-7, 6)		0.61<br>0.82

VAS: visual analogue scale; AUSCAN: Australian Canadian Osteoarthritis Hand Index; FIHOA: Functional Index for Hand Osteoarthritis; MHQ: Michigan Hand Outcome Questionnaire; NR: not reported

Supplementary Table 3. Overview of raw data for studies evaluating intra-articular corticosteroids for all outcomes at all time points

Study<br>Year<br>Country	Outcome	Outcome measures	End points	Intervention	Control	Mean difference (95% CI)/OR (95% CI)/RR (95% CI)	P value
**Ultrasound guided intra-articular methylprednisolone and lidocaine vs platelet-rich plasma**							
Malahias<br>(2021)<br>Greece	Pain	VAS	Baseline<br>3 months<br>12 months	70 (60-82.5)<br>20 (10.0-62.5)<br>65 (50-80)	75 (57.5-80.0)<br>40 (17.5-70.0)<br>20 (10.0-52.5)	- NR<br>- NR<br>- NR	0.76<br>0.46<br>0.015
	Disability	Q-DASH	Baseline<br>3 months<br>12 months	57.9 ± 25.6<br>32.6 ± 31.8<br>43.0 ± 27.6	50.4 ± 21.6<br>32.8 ± 29.2<br>20.4 ± 27.7	- NR<br>- NR<br>- NR	0.38<br>0.65<br>0.025
	Patient's satisfaction	Subjective (yes:no)	3 months<br>12 months	56%:44%<br>12.5%:87.5%	44%:56%<br>69%:31%	- NR<br>- NR	0.48<br>0.002
**Intra-articular triamcinolone acetate vs hyaluronic acid**							
Bahadir<br>(2009)<br>Turkey	Pain	VAS	Pretreatment<br>Month 1<br>Month 3<br>Month 6<br>Month 12	5.9±1.6<br>3.1±2.6<br>3.2±2.0<br>3.5±1.8<br>4.9±2.0	6.5±2.0<br>4.7±2.6<br>4.6±2.7<br>5.7±2.2<br>6.0±2.1	- NR<br>- NR<br>- NR<br>- NR<br>- NR	0.266<br>0.033<br>0.059<br>0.002<br>0.128
	Function	Duruöz Hand Index	Pretreatment<br>Month 1<br>Month 3<br>Month 6<br>Month 12	25.2±12.9<br>13.8±10.2<br>11.2±8.5<br>12.0±8.7<br>21.1±11.6	27.9±11.4<br>24.0±12.4<br>22.2±13.2<br>22.1±12.5<br>24.9±13.4	- NR	0.506<br>0.005<br>0.006<br>0.006<br>0.371
	Grip strength	Grip dynamometer	Pretreatment<br>Month 1<br>Month 3<br>Month 6<br>Month 12	38.0±9.5<br>45.2±11.5<br>42.0±9.8<br>41.6±10.0<br>39.7±12.3	32.0±8.4<br>35.6±12.9<br>36.2±10.8<br>35.2±10.8<br>34.9±11.1	- NR	0.060<br>0.034<br>0.099<br>0.091<br>0.291
	Tip Pinch strength	Pinch dynamometer	Pretreatment<br>Month 1<br>Month 3<br>Month 6<br>Month 12	8.9±1.5<br>8.9±2.1<br>9.0±2.0<br>8.6±2.0<br>8.2±1.9	6.8±1.7<br>7.0±2.1<br>7.3±1.9<br>7.5±2.1<br>7.1±2.0	- NR	NR
	Chunk Pinch strength	Pinch dynamometer	Pretreatment<br>Month 1<br>Month 3<br>Month 6<br>Month 12	10.3±1.9<br>10.5±2.4<br>10.6±2.4<br>10.0±2.3<br>9.9±2.2	8.1±2.0<br>8.4±2.8<br>8.6±2.3<br>8.8±2.4<br>8.5±2.3	- NR	NR
	Lateral Pinch strength	Pinch dynamometer	Pretreatment<br>Month 1<br>Month 3<br>Month 6<br>Month 12	13.1±2.3<br>13.3±2.9<br>13.4±3.1<br>12.9±2.8<br>12.7±2.7	10.2±2.4<br>10.7±3.3<br>11.1±3.0<br>11.1±3.2<br>14.9±16.7	- NR	NR
**Intra-articular triamcinolone vs sodium hyaluronate**							
Fuchs<br>(2006)<br>Germany	Pain	100mm VAS, Median values	Week1<br>Week 2<br>Week 3<br>Week 4<br>Week 5<br>Week 14<br>Week 26	63.5<br>61.5<br>46.0<br>33.0<br>20.0<br>22.0<br>45.5	65.5<br>64.5<br>54.0<br>41.0<br>34.0<br>35.0<br>30.0	- NR	NR
**Intra-articular sodium betamethasone sodium phosphate–betamethasone acetate vs Hylan G-F 20**							
Heyworth<br>(2008)<br>USA	Pain at rest	VAS	Baseline<br>2 weeks<br>4 weeks<br>12 weeks<br>26 weeks	5±1 (4.7#)<br>3.3 #<br>2.5 #<br>3.7 #<br>3.8 #	5±1 (4.6#)<br>3.1 #<br>3.9 #<br>3.1 #<br>3.2 #	- NR	NR
	Grip strength	Jamar dynamometer	Baseline<br>2 weeks<br>4 weeks<br>12 weeks<br>26 weeks	41 #<br>45 #<br>45 #<br>40 #<br>38 #	42 #<br>43 #<br>43 #<br>42 #<br>45 #	- NR	NR
	Disability	Q-DASH	Baseline<br>2 weeks<br>4 weeks	41 #<br>33 #<br>26 #	37 #<br>29 #<br>30 #	- NR	NR

			12 weeks<br>26 weeks	30 #<br>31 #	27 #<br>26 #		
	Lateral Pinch strength	Pinch dynamometer	Baseline<br>2 weeks<br>4 weeks<br>12 weeks<br>26 weeks	NR	NR	NR	NR
	Tip Pinch strength	Pinch dynamometer	Baseline<br>2 weeks<br>4 weeks<br>12 weeks<br>26 weeks	NR	NR	NR	NR
**Intra-articular methylprednisolone acetate vs dextrose**							
Jahangiri (2014) Iran	Pain intensity on pressure	VAS	Baseline<br>1 month<br>2 months<br>6 months	6.4 (1.8)<br>Around 2.8<br>Around 3.1<br>Around 3.5	6.7 (1.7)<br>Around 4.2<br>Around 2.9<br>Around 1.8		0.56
	Pain on joint movement	VAS	Baseline<br>1 month<br>2 months<br>6 months	4.5 (1.6)<br><br><br>2.4 (1.8)	5.0 (2.1)<br><br><br>1.2 (1.6)	 -0.7 (-1.8, 0.2)<br>1.0 (0.1, 2.0)<br>1.1 (0.2, 2.0)	0.29<br>0.14<br>0.02<br>0.02
	Pain threshold to pressure	Fischer's pressure algometer	Baseline<br>1 month<br>2 months<br>6 months				
	Pinch strength	Hydraulic pinch gauge	Baseline<br>1 month<br>2 months<br>6 months	11.6 (3.6)<br><br><br>12.7 (4.3)	9.6 (3.4)<br><br><br>11.9 (3.4)	 2.9 (0.9, 4.9)<br>1.1 (-0.8, 3.1)<br>0.8 (-1.3, 2.9)	0.03<br>0.005<br>0.25<br>0.45
	Hand function	HAQ-DI	Baseline<br>1 month<br>2 months<br>6 months	4.37 (1.4)<br><br><br>2.6 (1.5)	4.6 (1.8)<br><br><br>1.6 (1.3)	 0.5 (-1.3, 0.2)<br>1.0 (0.2, 1.9)<br>1.0 (0.2, 1.8)	0.63<br>0.15<br>0.01<br>0.01
**Intra-articular triamcinolone hexacetonide vs saline**							
Meenagh (2004) UK	Pain	VAS	Baseline<br>Changes from baseline at 4 weeks<br>Changes from baseline at 12 weeks<br>Changes from baseline at 24 weeks	52 (40 to 72)<br>10.5 (-8.0 to 12.6)<br>3.5 (-8.5 to 4.9)<br>0.0 (-12.5 to 2.3)	56 (50 to 78)<br>18.5 (3.5 to 20.1)<br>23.3 (6.0 to 29.3)<br>14.0 (-12.5 to 16.9)	NR	
	Stiffness	In minutes	Baseline<br>Changes from baseline at 4 weeks<br>Changes from baseline at 12 weeks<br>Changes from baseline at 24 weeks	20 (0 to 30)<br>0.0 (-5.0 to 2.5)<br>0.0 (-7.5 to 1.3)<br>0.0 (-7.5 to 1.0)	15 (10 to 30)<br>2.5 (0.0 to 3.0)<br>2.5 (-10.0 to 3.8)<br>5.0 (-5.0 to 8.5)	NR	
	Tenderness	Single fingertip pressure	Baseline<br>Changes from baseline at 4 weeks<br>Changes from baseline at 12 weeks<br>Changes from baseline at 24 weeks	2 (1 to 3)<br>0 (-1.0 to 0.9)<br>0.5 (-1.0 to 1.0)<br>0.5 (-1.5 to 1.0)	2 (1 to 2)<br>1 (0.0 to 1.3)<br>2 (-1.5 to 3.1)<br>2.5 (-1.5 to 3.5)	NR	
**Ultrasonography guided intra-articular betamethasone vs hyaluronic acid**							
Monfort (2014) Spain	Function	FIOHA	Baseline<br>Day 7<br>Day 14<br>Day 30<br>Day 90	11.5 [IQR 8-14]<br>-1 (-2 and -1)<br>-1 (-4 and -0)<br>-3 (-7.5 and -0)<br>-1 (-3 and -1)	11.0 [IQR7-14.7]<br>0 (-3 and -1)<br>-2 (-5 and -0)<br>-3 (-6.7 and -0)<br>-4 (-8 and -1)	NR	0.814

			Day 180	-1 (-3 and -3)	-3 (-8.7 and -1)		
	Pain	VAS	Baseline<br>Day 7<br>Day 14<br>Day 30<br>Day 90<br>Day 180	6.4 (1.3)<br>-0.95 (1.60)<br>-2.01 (1.84)<br>-2.53 (2.26)<br>-1.55 (2.14)<br>-1.42 (2.35)	6.0 (1.8)<br>-0.71 (1.66)<br>-1.42 (2.23)<br>-1.97 (2.62)<br>-1.61 (2.53)<br>-1.97 (2.73)	NR	0.171
**Intra-articular triamcinolone plus lidocaine vs lidocaine**							
Spolidoro (2015) Brazil	Pain at rest	VAS	Baseline<br>Week 1<br>Week 4<br>Week 8<br>Week 12	6.1 (1.7)<br>2.6 (2.9)<br>1.3 (2.1)<br>1.4 (2.6)<br>0.8 (1.7)	6.1 (1.6)<br>1.7 (2.7)<br>1.6 (2.6)<br>1.6 (2.6)<br>0.9 (2.2)	NR	
	Pain	AUSCAN	Baseline<br>Week 1<br>Week 4<br>Week 8<br>Week 12	8.8 (4.8)<br>7.0 (4.6)<br>5.9 (4.9)<br>6.0 (4.9)<br>5.3 (4.7)	9.2 (4.3)<br>8.3 (5.1)<br>7.8 (5.4)<br>8.3 (4.5)<br>7.0 (4.8)	NR	
	Pain at movement	VAS	Baseline<br>Week 1<br>Week 4<br>Week 8<br>Week 12	6.5 (1.8)<br>3.9 (3.1)<br>2.8 (2.9)<br>1.8 (2.6)<br>2.2 (2.9)	6.6 (1.4)<br>4.1 (2.9)<br>3.0 (3.0)<br>4.0 (3.3)<br>4.0 (3.2)	NR	
	Swollen joint	VAS (physician assessed)	Baseline<br>Week 1<br>Week 4<br>Week 8<br>Week 12	3.0 (1.5)<br>2.0 (1.5)<br>1.4 (1.4)<br>0.7 (0.8)<br>1.1 (1.2)	3.0 (1.7)<br>2.1 (1.4)<br>2.0 (1.2)<br>1.8 (1.3)<br>2.0 (1.3)	NR	
	Grip strength	Jamar dynamometer	Baseline<br>Week 1<br>Week 4<br>Week 8<br>Week 12	14.85 (6.71)<br>14.12 (6.56)<br>15.09 (6.57)<br>15.52 (7.33)<br>16.21 (6.24)	13.68 (7.59)<br>13.7 (7.79)<br>14.65 (7.78)<br>15.44 (7.58)<br>15.23 (7.70)	NR	
	Pinch strength	Pinch gauge dynamometer	Baseline<br>Week 1<br>Week 4<br>Week 8<br>Week 12	6.12 (1.82)<br>5.95 (1.84)<br>6.36 (1.61)<br>6.39 (1.98)<br>6.50 (1.88)	5.79 (2.40)<br>6.27 (1.86)<br>6.35 (2.11)<br>6.47 (1.64)<br>6.24 (1.75)	NR	
	Function	AUSCAN	Baseline<br>Week 1<br>Week 4<br>Week 8<br>Week 12	15.4 (10.4)<br>14.6 (9.5)<br>12.9 (8.9)<br>12.7 (9.5)<br>12.3 (9.8)	17.9 (8.9)<br>17.8 (9.7)<br>15.9 (8.9)<br>15.8 (9.7)<br>16.7 (9.8)	NR	
	Stiffness	AUSCAN	Baseline<br>Week 1<br>Week 4<br>Week 8<br>Week 12	1.7 (1.4)<br>1.1 (1.3)<br>1.2 (1.5)<br>1.6 (1.4)<br>1.2 (1.4)	2.0 (1.5)<br>1.5 (1.5)<br>1.8 (1.5)<br>1.9 (1.2)<br>2.0 (1.4)	NR	
	Total	AUSCAN GLOBAL	Baseline<br>Week 1<br>Week 4<br>Week 8<br>Week 12	25.9 (15.1)<br>22.7 (13.8)<br>20.0 (13.9)<br>20.3 (14.6)<br>18.8 (14.1)	29.1 (13.4)<br>27.5 (15.2)<br>25.5 (14.0)<br>26.0 (14.0)<br>25.7 (14.4)	NR	
**Intra-articular depot methylprednisolone acetate vs hyaluronic acid**							
Stahl (2005) Israel	Pain at rest	VAS	Baseline<br>Month 1<br>Month 3<br>Month 6	4.2 (2.2-6.4)<br>-1.8±2.0<br>-1.9±1.8<br>-2.2±2.0	4.5 (2.4-6.8)<br>-2.2±2.0<br>-2.0±2.0<br>-2.2±2.1	NR	
	Pain at rest	VAS	Baseline<br>Month 1<br>Month 3<br>Month 6	4.2 (2.2-6.4)<br>2 (0.2-4)<br>2.1 (0-4.5)#<br>2 (0.1-4)	4.5 (2.4-6.8)<br>3 (0.8-5.2)<br>2.5 (0.1-5)<br>2.2 (0.2-4.5)	NR	
	Pain at activity	VAS	Baseline<br>Month 1<br>Month 3<br>Month 6	7.7<br>-2.0±2.3<br>-2.5±2.0<br>-2.7±2.2	7.9<br>-1.9±1.8<br>-2.2±1.8<br>-2.2±1.9	NR	
	Grip strength	Grasp dynamometer	Baseline<br>Month 1<br>Month 3<br>Month 6	19.8 (avg)<br>20.3<br>20.9<br>21.3	19.7<br>19.8<br>20.6<br>21.1	NR	

	Pinch strength (lateral)	pinch dynamometer	Baseline<br>Month 1<br>Month 3<br>Month 6	5.4<br>5.7<br>5.7<br>5.8	5.2<br>5.4<br>5.5<br>5.7	NR	
**Intra-articular betamethasone vs hyaluronic acid**							
Sabaah (2020)a Egypt	Tenderness grading		Baseline<br>1<br>2<br>3<br>Month 1<br>0<br>1<br>2<br>Month 3<br>0<br>1<br>2<br>3	 3 (20%)<br>9 (60%)<br>3 (20%)<br><br>9 (60%)<br>5 (33.3%)<br>1 (6.7%)<br><br>1 (6.7%)<br>5 (33.3%)<br>8 (53.3%)<br>1 (6.7%)	 3 (20%)<br>10 (66.7%)<br>2 (13.3%)<br><br>6 (40%)<br>9 (60%)<br>0 (0%)<br><br>12 (80%)<br>2 (13.3%)<br>1 (6.7%)<br>0 (0%)	NR	
	Pain	VAS	Baseline<br>Month 1<br>Month 3	7 (IQR 6-8)<br>4 (3-5)<br>5 (5-8)	7 (5-8)<br>4 (3-5)<br>3 (2-3)	NR	<0.001
	Total score	AUSCAN	Baseline<br>Month 1<br>Month 3	33 (29-35)<br>25 (21-26)<br>33 (28-33)#	33 (26-39)<br>22 (18-26)<br>20 (17-23)#	NR	
	Functional score	AUSCAN functional subscale	Baseline<br>Month 1<br>Month 3	22 (20-25)<br>16 (14-20)<br>22 (20-23)	23 (20-25)<br>16 (14-18)<br>14 (12-16)	NR	
	Grip strength	Digital hand dynamometer	Baseline<br>Month 1<br>Month 3	15#<br>21#<br>16#	15#<br>23#<br>26#	NR	
	Pinch strength	Digital hand dynamometer	Baseline<br>Month 1<br>Month 3	3.7#<br>5.5#<br>3.7#	3.2#<br>5.6#<br>6.5#	NR	
	Grind test		Baseline<br>Month 1<br>Month 3	NR	NR		
	Lever test		Baseline<br>Month 1<br>Month 3	NR	NR		
**Intra-articular betamethasone vs platelet-rich plasma**							
Sabaah (2020)b Egypt	Tenderness grading		Baseline<br>1<br>2<br>3<br>Month 1<br>0<br>1<br>2<br>Month 3<br>0<br>1<br>2<br>3	 3 (20%)<br>9 (60%)<br>3 (20%)<br><br>9 (60%)<br>5 (33.3%)<br>1 (6.7%)<br><br>1(6.7%)<br>5 (33.3%)<br>8 (53.3%)<br>1 (6.7%)	 5 (33%)<br>7 (46.7%)<br>3 (20%)<br><br>10 (66.7%)<br>4 (26.7%)<br>1 (6.7%)<br><br>0 (0%)<br>6 (40%)<br>9 (60%)<br>0 (0%)	NR	
	Pain	VAS	Baseline<br>Month 1<br>Month 3	7 (IQR 6-8)<br>4 (3-5)<br>5 (5-8)	8 (6-8)<br>4 (3-5)<br>5 (4-6)	NR	0.058
	Total score	AUSCAN	Baseline<br>Month 1<br>Month 3	33 (29-35)<br>25 (21-26)<br>33 (28-33)#	35 (32-37)<br>28 (26-30)<br>26 (25-28)#	NR	
	Functional score	AUSCAN functional subscale	Baseline<br>Month 1<br>Month 3	22 (20-25)<br>16 (14-20)<br>22 (20-23)	24 (22-25)<br>20 (18-20)<br>21 (18-22)	NR	
	Grip strength	Digital hand dynamometer	Baseline<br>Month 1<br>Month 3	15#<br>21#<br>16#	16#<br>17#<br>16#	NR	
	Pinch strength	Digital hand dynamometer	Baseline<br>Month 1<br>Month 3	3.7#<br>5.5#<br>3.7#	4.2#<br>4.4#<br>4.3#	NR	
	Grind test		Baseline<br>Month 1	NR	NR		

			Month 3				
	Lever test		Baseline<br>Month 1<br>Month 3	NR	NR		

#derived from graph; VAS: visual analogue scale; AUSCAN: Australian Canadian Osteoarthritis Hand Index; HAQ-DI: Health Assessment Questionnaire Disability Index Questionnaire; FIHOA: Functional Index for Hand Osteoarthritis; MHQ: Michigan Hand Outcome Questionnaire; Q-DASH: Quick Disabilities of the Arm, Shoulder and Hand questionnaire; NR: not reported; NA: Not applicable; Sabaah 2020(a): corticosteroid vs hyaluronic acid; Sabaah 2020(b) corticosteroid vs plasma-rich plasma; MD: mean difference; CI: confidence interval; OR: odds ratio; RR: risk ratio

Supplementary Table 4. Search of clinical trial registers and registries for trials with Completed or Unknown status that are not published

Identifier	Title	Status	Actual/estimated completion date
NCT03102788	Task Shifting in the care for patients with hand Osteoarthritis	Completed	November 2020
NCT00398866	A Study of Hyaluronan for the Treatment of Osteoarthritis in the Thumb	Completed	March 2013
ISRCTN63038599	Comparing sodium hyaluronate injection into the carpo-metacarpal joint of the thumb with steroid in the treatment of carpo-metacarpal osteoarthritis	Completed	June 2005

Supplementary Table 5. Rob Me assessment for random effect meta-analysis of the effect of oral corticosteroid vs placebo on pain at short term (4-6 weeks)

**Details of the synthesis being assessed for risk of bias**		
**Specify the synthesis**	Random effect meta-analysis of the effect of oral corticosteroid vs placebo on pain at short term (4-6 weeks)	
**Specify the synthesized result (e.g. estimate and 95% CI)**	-0.53 (-0.79 to -0.28)	
**Specify the number of included studies and participants**	3, 245	
**Risk of bias assessment**		
**Signalling questions**	**Comments**	**Response options**
The following questions relate to the within-study assessment of non-reporting bias ('known unknowns')		
**4.1. Of the studies identified, was there any for which no result was available for inclusion in the synthesis, likely because of the P value, magnitude or direction of the result generated (refer to Step 2)?**	N	Y / N
**4.2. If Y to 4.1: Is it likely that there would be a notable change to the synthesized effect estimate if the omitted results had been included?**		NA / Y / PY / PN / N / NI
**4.3. Of the studies identified, was there any for which it was unclear whether an eligible result was generated (refer to Step 2)?**	N (3 studies evaluating oral corticosteroid has result for pain at 4-6 weeks)	Y / N
**4.4. If Y to 4.3: Is it likely that there would be a notable change to the synthesized effect estimate if the potentially omitted results had been included?**		NA / Y / PY / PN / N / NI
The following questions relate to the across-study assessment of non-reporting bias ('unknown unknowns')		
**4.5 Do circumstances indicate potential for some eligible studies not being identified because of the P value, magnitude or direction of the results generated (refer to Step 3)?**	Y	Y / PY / PN / N
**4.6. If Y/PY to 4.5: Is it likely that studies not identified had results that were eligible for inclusion in the synthesis?**	PY	NA / Y / PY / PN / N
**4.7. If Y to 4.1 or 4.3 or Y/PY to 4.5: Does the pattern of observed study results suggest that the synthesis is likely to be missing results that were systematically different (in terms of P value, magnitude or direction) from those observed?**	N ((as they all do not have statistically significant results)	NA / Y / PY / PN / N

**4.8. <u>If Y/PY/NI to 4.2, 4.4, 4.6 or 4.7:</u> Did sensitivity analyses suggest that the synthesized result was biased due to missing results?**	PN	NA / Y / PY / <u>PN / N</u>
**Risk of bias judgement**	Some concerns	Low / High / Some concerns
Optional: What is the predicted direction of bias for this synthesis?		NA / Favours experimental / Favours comparator / Towards null /Away from null / Unpredictable

Supplementary Table 6. Rob Me assessment for random effect meta-analysis of the effect of intra-articular corticosteroid vs placebo on pain at short term (4-6 weeks)

**Details of the synthesis being assessed for risk of bias**		
**Specify the synthesis**	Random effect meta-analysis of the effect of IA corticosteroid vs placebo on pain at short term (4-6 weeks)	
**Specify the synthesized result (e.g. estimate and 95% CI)**	0.22 (-1.17 to 1.61)	
**Specify the number of included studies and participants**	4, 189	
**Risk of bias assessment**		
**Signalling questions**	**Comments**	**Response options**
The following questions relate to the within-study assessment of non-reporting bias ('known unknowns')		
**4.1. Of the studies identified, was there any for which no result was available for inclusion in the synthesis, likely because of the P value, magnitude or direction of the result generated (refer to Step 2)?**	N	Y / N
**4.2. If Y to 4.1: Is it likely that there would be a notable change to the synthesized effect estimate if the omitted results had been included?**		NA / Y / PY / PN / N / NI
**4.3. Of the studies identified, was there any for which it was unclear whether an eligible result was generated (refer to Step 2)?**	Y (one NI regarding protocol)	Y / N
**4.4. If Y to 4.3: Is it likely that there would be a notable change to the synthesized effect estimate if the potentially omitted results had been included?**	PN (single study with 33 participants so less chance to influence result)	NA / Y / PY / PN / N / NI
The following questions relate to the across-study assessment of non-reporting bias ('unknown unknowns')		
**4.5 Do circumstances indicate potential for some eligible studies not being identified because of the P value, magnitude or direction of the results generated (refer to Step 3)?**	Y	Y / PY / PN / N
**4.6. If Y/PY to 4.5: Is it likely that studies not identified had results that were eligible for inclusion in the synthesis?**	PY	NA / Y / PY / PN / N
**4.7. If Y to 4.1 or 4.3 or Y/PY to 4.5: Does the pattern of observed study results suggest that the synthesis is likely to be missing results that were systematically different (in terms of P value, magnitude or direction) from those observed?**	N (as they all do not have statistically significant results)	NA / Y / PY / PN / N
**4.8. If Y/PY/NI to 4.2, 4.4, 4.6 or 4.7: Did sensitivity analyses suggest that the synthesized result was biased due to missing results?**	PN	NA / Y / PY / PN / N
**Risk of bias judgement**	Some concerns	Low / High / Some concerns

Optional: What is the predicted direction of bias for this synthesis?		NA / Favours experimental / Favours comparator / Towards null /Away from null / Unpredictable

Supplementary Table 7. GRADE assessment for random effect meta-analysis of the effect of oral corticosteroid vs placebo on pain at short term (4-6 weeks)

	Risk of bias	Indirectness	Inconsistency	Imprecision	Reporting bias	Score	Grade rating
	Are there limitations with the study methods?	Do the results not really apply to my question?	Are the results inconsistent across studies?	Are there too few people or events?	Are we missing studies or have selective studies?	Total 4	
	Serious (-1)	none	Serios (-1)	serious (-1)	Not serious	4-1-1-1=1	low
	(as one study high risk with 30% weight)	-similar to our question<br>-similar to our inclusion criteria	I2 = 73%	(too few participants 245 in total and CI same side (-0.79 to -0.28)	Some concern in –ROB-ME		

Supplementary Table 8. GRADE assessment for random effect meta-analysis of the effect of intra-articular corticosteroid vs placebo on pain at short term (4-6 weeks)

	Risk of bias	Indirectness	Inconsistency	Imprecision	Reporting bias	Score	Grade rating
	Are there limitations with the study methods?	Do the results not really apply to my question?	Are the results inconsistent across studies?	Are there too few people or events?	Are we missing studies or have selective studies?		
	Serious (-1)	none	Serious (-1)	serious (-1)	Not serious	4-1-1-1=1	low
	One with 14% high risk<br>Rest 2: low risk	-similar to our question<br>-similar to our inclusion criteria	I2 =78%	(too few participants 159 in total and CI same side -1.34 to -0.11) and not between harm and benefit	Some concern in ROB-ME assessment		

Supplementary Figure 1. Risk of bias assessment using RoB 2 tool considering patient reported pain and functional outcome

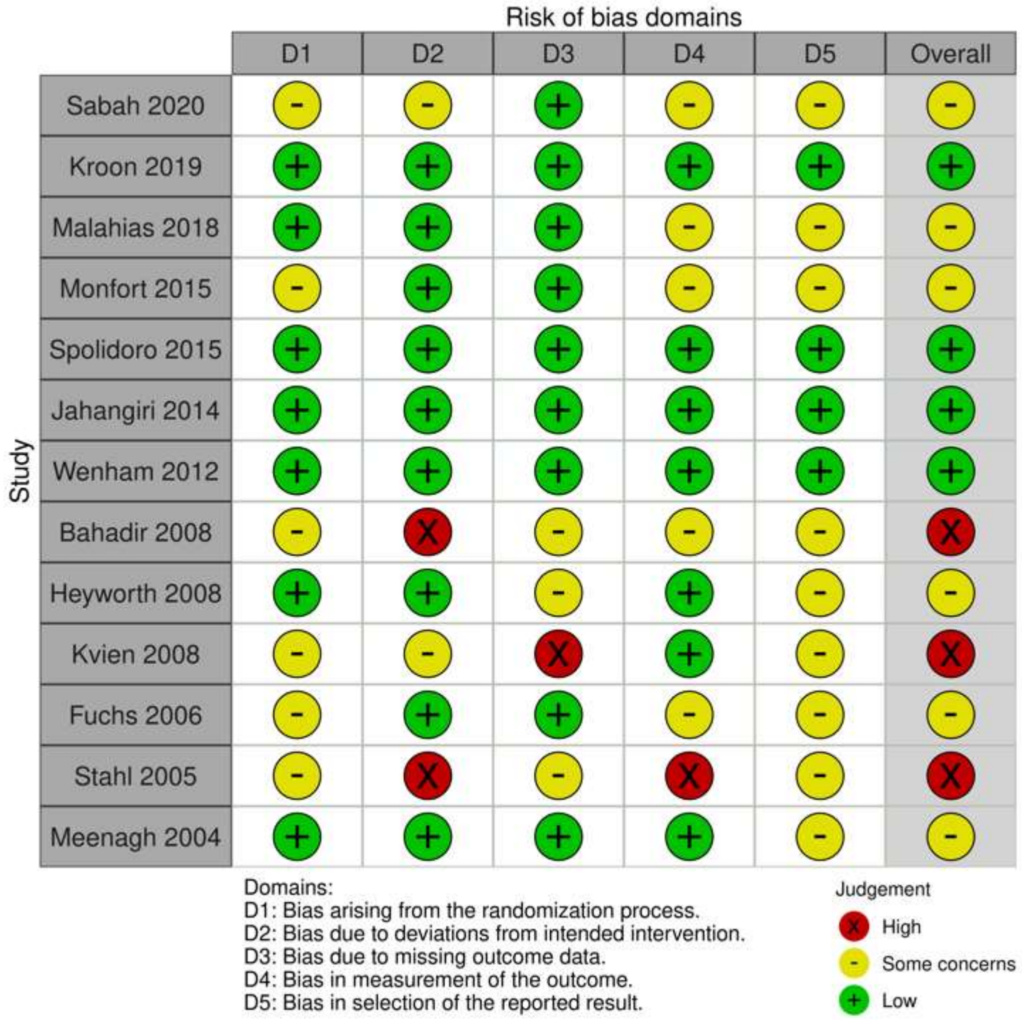

Supplementary Figure 2. Random effects meta-analysis of the standard mean difference in stiffness, based on oral corticosteroid or placebo at 4-6 weeks

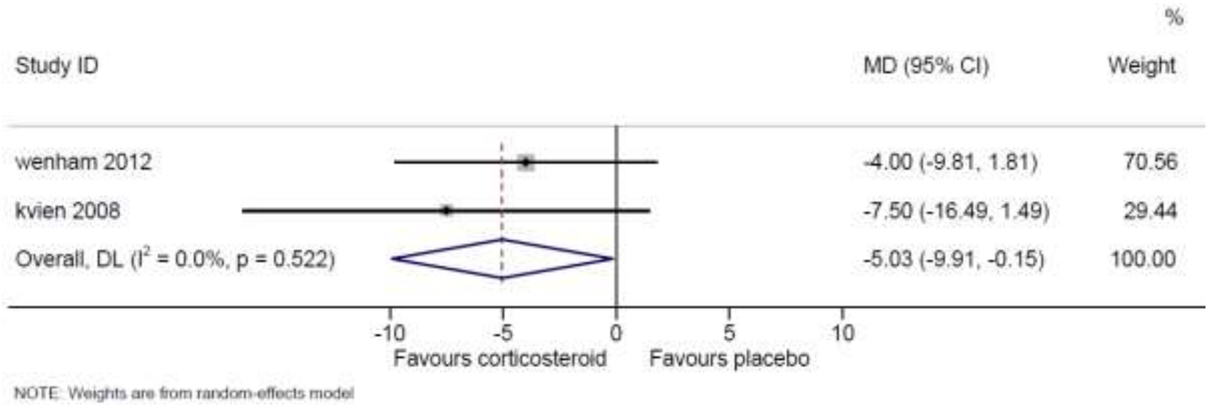

Supplement: Supplementary file 1 — Additional file 1: Supplementary Table 1. Search strategy for systematic review in Ovid MEDLINE(R) and Epub Ahead of Print, In-Process, In-Data-Review & Other Non-Indexed Citations, Daily and Versions(R)/Embase/Cochrane, Ovid Embase Classic+Embase, Ovid EBM Reviews - Cochrane Central Register of Controlled Trials. Supplementary Table 2. Overview of raw data for studies evaluating oral corticosteroids for all outcomes at all time points. Supplementary Table 3. Overview of raw data for studies evaluating intra-articular corticosteroids for all outcomes at all time points. Supplementary Table 4. Search of clinical trial registers and registries for trials with Completed or Unknown status that are not published. Supplementary Table 5. Rob Me assessment for random effect meta-analysis of the effect of oral corticosteroid vs placebo on pain at short term (4-6 weeks). Supplementary Table 6. Rob Me assessment for random effect meta-analysis of the effect of intra-articular corticosteroid vs placebo on pain at short term (4-6 weeks). Supplementary Table 7. GRADE assessment for random effect meta-analysis of the effect of oral corticosteroid vs placebo on pain at short term (4-6 weeks). Supplementary Table 8. GRADE assessment for random effect meta-analysis of the effect of intra-articular corticosteroid vs placebo on pain at short term (4-6 weeks). Supplementary Figure 1. Risk of bias assessment using RoB 2 tool considering patient reported pain and functional outcome. Supplementary Figure 2. Random effects meta-analysis of the standard mean difference in stiffness, based on oral corticosteroid or placebo at 4-6 weeks. [file 12891_2022_5619_MOESM1_ESM.pdf]
